# Targeting VEGF-A in myeloid cells enhances natural killer cell responses to chemotherapy and ameliorates cachexia

**DOI:** 10.1038/ncomms12528

**Published:** 2016-08-19

**Authors:** Ralph Klose, Ewelina Krzywinska, Magali Castells, Dagmar Gotthardt, Eva Maria Putz, Chahrazade Kantari-Mimoun, Naima Chikdene, Anna-Katharina Meinecke, Katrin Schrödter, Iris Helfrich, Joachim Fandrey, Veronika Sexl, Christian Stockmann

**Affiliations:** 1 Institut National de la Santé et de la Recherche Médicale (INSERM), Paris Cardiovascular Research Center, Unit 970, 56 Rue Leblanc, 75015 Paris, France; 2 Institute of Pharmacology and Toxicology, University of Veterinary Medicine Vienna, 1210 Vienna, Austria; 3 Institut für Physiologie, Universitätsklinikum Essen, Universität Duisburg-Essen, 45147 Essen, Germany; 4 Skin Cancer Unit of the Department of Dermatology, West German Cancer Center, University of Duisburg-Essen, Hospital Essen, DKTK Essen, 45147 Essen, Germany

## Abstract

Chemotherapy remains a mainstay of cancer treatment but its use is often limited by the development of adverse reactions. Severe loss of body weight (cachexia) is a frequent cause of death in cancer patients and is exacerbated by chemotherapy. We show that genetic inactivation of vascular endothelial growth factor (VEGF)-A in myeloid cells prevents chemotherapy-induced cachexia by inhibiting skeletal muscle loss and the lipolysis of white adipose tissue. It also improves clearance of senescent tumour cells by natural killer cells and inhibits tumour regrowth after chemotherapy. The effects depend on the chemoattractant chemerin, which is released by the tumour endothelium in response to chemotherapy. The findings define chemerin as a critical mediator of the immune response, as well as an important inhibitor of cancer cachexia. Targeting myeloid cell-derived VEGF signalling should impede the lipolysis and weight loss that is frequently associated with chemotherapy, thereby substantially improving the therapeutic outcome.

## Abstract (web summary)

Chemerin is an adipokine often downregulated in tumours. Here the authors show that chemotherapy induces chemerin production by endothelial cells, leading to cachexia, and that VEGF ablation in myeloid cells prevents cachexia in a chemerin-dependent manner, and improves chemotherapeutic effects.

## Graphical abstract

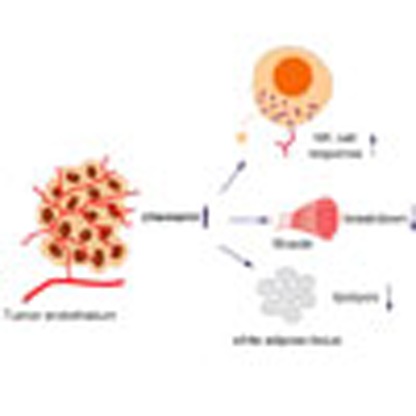

Despite its frequent side effects, chemotherapy generally represents the first course of treatment for cancer patients. The benefits of chemotherapeutic agents stem not only from direct effects on the tumour cell but also from influences on the tumour microenvironment, resulting in a robust immune response that can be crucial to the therapeutic outcome^1^. However, drug delivery poses a significant problem as the vasculature of tumours is inefficient^2^. In most tumours, despite high vascular density, the vasculature differs from normal vascular networks and is characterized by an inefficient blood supply. Vessel abnormalities include increased permeability and tortuosity, as well as decreased pericyte coverage, which frequently cause scarce delivery of chemotherapy to the tumour and tumour hypoxia as well. Therefore, strategies to reverse this phenotype and to ‘normalize' the tumour vasculature have gained increasing interest^2^. Using mouse models, we have shown that specific deletion of vascular endothelial growth factor (VEGF) in tumour-infiltrating myeloid cells leads to normalized tumour blood vessels and increased tumour cell apoptosis^3^.

Cancer-induced cachexia is the immediate cause of death in ∼15% of cancer patients^4^,^5^,^6^. It is characterized by involuntary weight loss that is resistant to nutritional supplementation^7^. Weight loss starts with degradation of skeletal muscle and the breakdown of white adipose tissue (WAT) mediated by the lipolytic enzymes adipose triglyceride lipase (Atgl) and hormone-sensitive lipase (Hsl)^8^. Cachexia is believed to be induced by tumour-derived factors, such as tumour necrosis factor-α (TNF-α) and interleukin (IL)-6 (refs 9, 10). After an initial reduction of tumour mass, treatment with chemotherapeutic agents frequently exacerbates cachexia, hampering further treatment and increasing mortality^11^,^12^. There is an urgent need for treatment regimens that counter the development of cachexia and thus allow continued chemotherapy.

Chemerin was initially defined as an adipokine^13^ but has received considerable interest as a chemoattractant for macrophages, dendritic cells and natural killer (NK) cells^14^,^15^,^16^. NK cells and cytotoxic T cells are particularly important in the immunosurveillance and suppression of tumours^17^,^18^, and chemerin has been shown to improve NK cell-based tumour surveillance. Expression of the chemerin gene ((*Rarres (retinoic acid receptor responder) 2*) is frequently downregulated in human solid tumours, including lung cancer and melanoma. Overexpression of chemerin in melanoma cells in mouse models results in increased NK cell recruitment and tumour suppression^19^.

We now show that chemerin is a pivotal regulator of the chemotherapy-elicited immune response, as well as of therapy-associated cachexia. We demonstrate further that endothelial release of chemerin on chemotherapy can be enhanced by targeting VEGF-A in myeloid cells, leading to improved chemotherapeutic success.

## Results

### Targeting of VEGF-A in myeloid cells delays tumour growth

**Figure 1:**
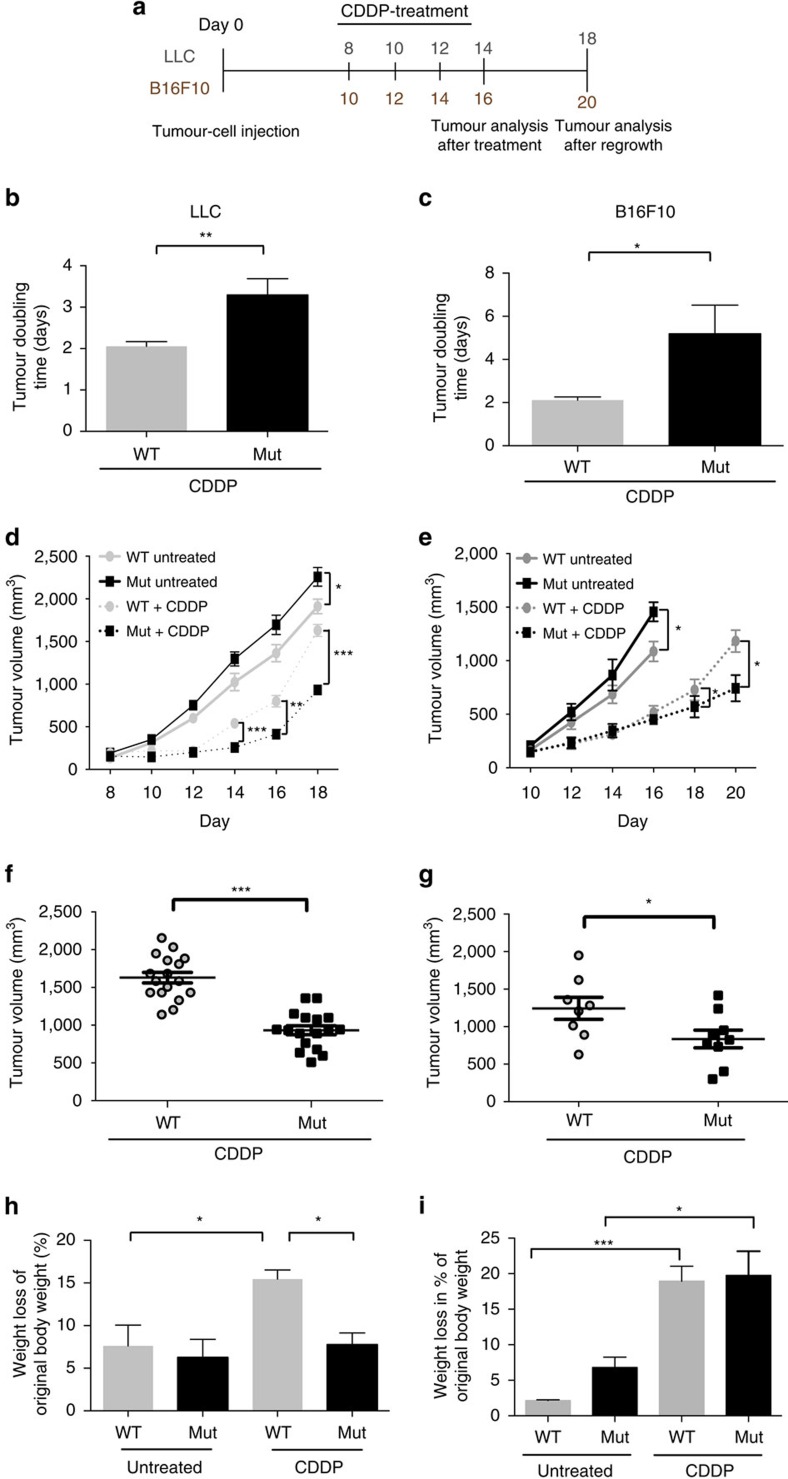
Loss of myeloid cell VEGF-A delays tumour growth and ameliorates cancer cachexia after chemotherapy. (**a**) Schematic representation of experimental procedure to study tumour regrowth of LLC and B16F10 isografts in WT and mutant (Mut, LysMCre/VEGF^f^/^f^) mice. Cells (10^7^) of the tumour cell line were subcutanously (s.c.) injected into mice and 8 mg kg^−1^ cisplatin (CDDP) was administered by i.p. at the indicated time points. Tumours were allowed to grow until the maximum size was reached. (**b**) Determination of speed of tumour growth of LLC isografts after chemotherapy. Tumour doubling time was calculated from the last treatment and the end volume for a total period of 6 days (WT, *n*=17; Mut, *n*=17). (**c**) Determination of speed of tumour growth of B16F10 isografts after chemotherapy. Tumour doubling time was calculated from the last treatment and the end volume for a total period of 6 days (WT, *n*=8; Mut, *n*=8). (**d**) Graphical representation of tumour growth kinetics of untreated and cisplatin-treated LLC isografts after s.c. injection of tumour cells into different cohorts of mice (WT untreated, *n*=9; Mut untreated, *n*=11; WT+CDDP, *n*=17; Mut+CDDP, *n*=17). (**e**) Graphical representation of tumour growth and regrowth kinetics of cisplatin-treated B16F10 isografts after s.c. injection of tumour cells (WT untreated, *n*=6; Mut untreated, *n*=6; WT+CDDP, *n*=8; Mut+CDDP, *n*=8). (**f**) Tumour volumes of cisplatin-treated LLC isografts at endpoint day 18 (WT+CDDP, *n*=17; Mut+CDDP, *n*=17). (**g**) Tumour volumes of cisplatin-treated B16F10 isografts at endpoint day 18 (*n*≥9). (**h**) Body weight loss of untreated and cisplatin-treated LLC-bearing mice at endpoint day 18. Weight loss is given as percentage of the original body weight (WT: *n*≥4; Mut: *n*≥7). (**i**) Body weight loss of untreated and cisplatin-treated B16F10-bearing mice at endpoint. Weight loss is given as percentage of the original body weight (day 16 untreated, day 20 treated; WT: *n*≥5; Mut: *n*≥7). Bars represent mean values; error bars indicate s.e.m.; statistical significance was determined by an unpaired Student's *t*-test for two samples or by one-way analysis of variance followed by Bonferroni *post-hoc* test when more than two groups were compared. Statistical significance is indicated as *P<0.05, **P<0.01 and ***P<0.001.

We have previously crossed mice with a *loxP*-flanked *Vegfa* allele to mice with the Cre recombinase under the control of the lysozyme M promoter. The *VEGF-A* gene is specifically deleted in the myeloid cells of the resulting mutant (Mut, LysMCre/VEGF^f^/^f^) mice and the animals' response to chemotherapy is improved: the mice show vascular normalization and an increase in tumour cell apoptosis^3^. We subjected wild-type (WT, LysMCre−/VEGF+/+) and mutant mice carrying Lewis lung carcinomas (LLCs) or B16F10 (B16) melanomas to three cycles of cisplatin treatment (*cis*-diamminedichloridoplatinum(II) (cisplatin, CDDP), 8 mg per kg body weight, see scheme Fig. 1a). In LLC and B16 tumours, loss of VEGF-A in myeloid cells significantly increased tumour-doubling time (Fig. 1b for LLC and Fig. 1c for B16) and was associated with significantly reduced endpoint tumour volumes (Fig. 1d,f for LLC and Fig. 1e,g for B16). In contrast, WT tumours reached endpoint volumes comparable to those of untreated tumours (Fig. 1d,e), indicative of treatment failure. Ulcerations in the mice injected with B16 melanoma cells forced termination of the control experiment ahead of schedule (Fig. 1e).

Treatment with cytotoxic agents frequently exacerbates cachexia and limits the outcome of therapy^11^,^12^. Untreated LLC- and B16-bearing WT and Mut mice had similarly reduced body weights at endpoint (Fig. 1h,i, respectively). On chemotherapy with cisplatin, the loss of body weight in the LLC (Fig. 1h) but not in the B16 model (Fig. 1i) depended on the presence of myeloid VEGF-A. LLC-bearing WT mice showed a significant drop in body weight that was mitigated in Mut mice by deletion of myeloid cell-derived VEGF-A (Fig. 1h).

### Deletion of myeloid-derived VEGF-A improves drug delivery

**Figure 2:**
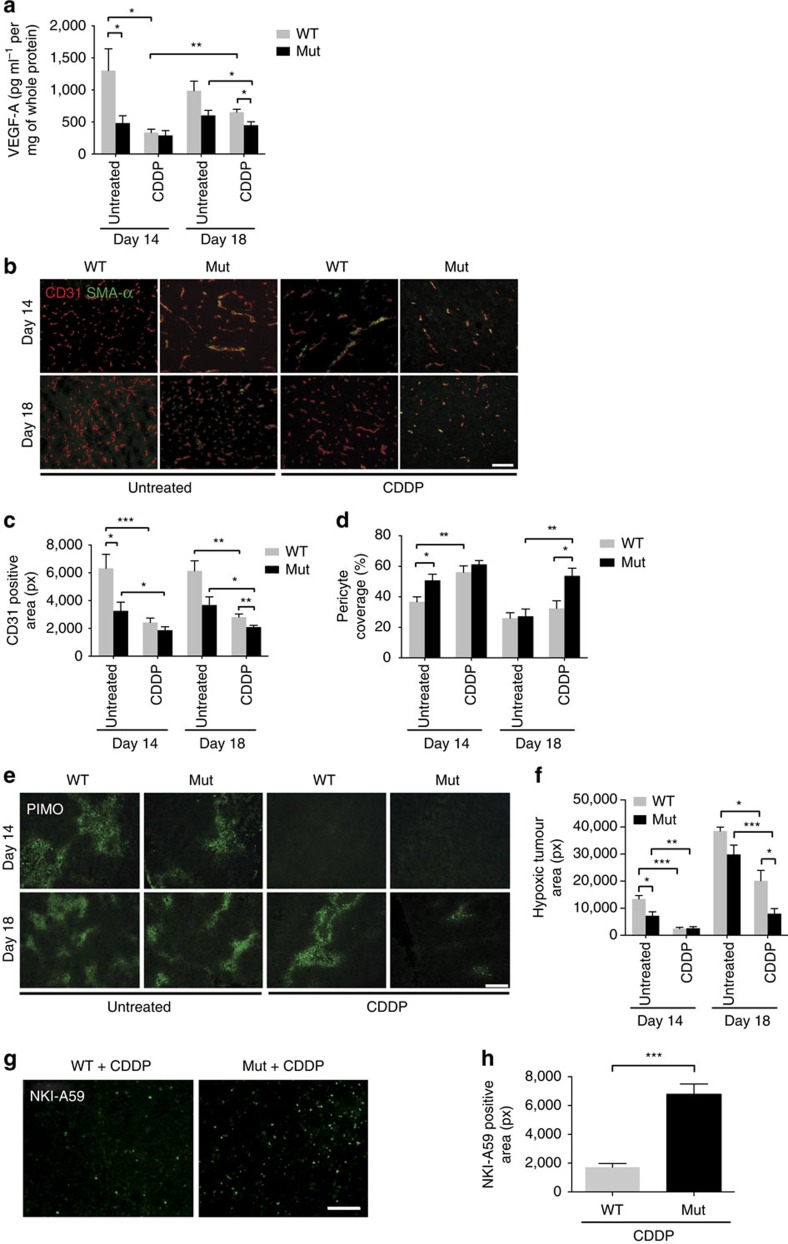
Targeting myeloid cell VEGF-A induces sustained vascular normalization. (**a**) Determination of levels of VEGF protein in LLC tumour lysates from WT and mutant (mut, LysMCre/VEGF^f^/^f^) animals by ELISA (WT, *n*≥4; Mut, *n*≥4). (**b**) Representative photomicrographs of co-immunolabelled CD31 and SMA-α LLC tumour sections derived from WT and mutant mice. (**c**) Quantitative analysis of CD31 immunostaining shown in **b** (untreated, *n*>5; CDDP, *n*>6). (**d**) Quantification of pericyte coverage as assessed by co-localization studies in **b**. The fraction of pericyte coverage is given as the ratio of the number of SMA-α- to the number of CD31-positive cells (untreated, *n*=4; CDDP, *n*>5). (**e**) Microscopic images representing the degree of tumour hypoxia in WT and mutant animals under designated conditions. Pimonidazole (60 mg kg^−1^) was given by i.p. injection 1 h before killing and detected by the monoclonal antibody Mab-1. (**f**) Quantification of pimodinazole-positive areas in **e** (WT control, *n*=3; Mut control, *n*=5; WT+CDDP, *n*≥12; Mut+CDDP, *n*≥10). (**g**) Visualization of cisplatin–DNA adducts in WT and Mut tumour sections by immunofluorescent staining (day 14). (**h**) Quantitative analysis of **g** (*n*=5). Bars represent mean values; error bars indicate the s.e.m.; statistical significance was determined by an unpaired Student's *t*-test for two samples or by one-way analysis of variance followed by Bonferroni *post-hoc* test when more than two groups were compared. Statistical significance is indicated as *P<0.05, **P<0.01 and ***P<0.001. Scale bar, 100 μm.

Irrespective of the genotype, cisplatin treatment reduced levels of VEGF-A, lowered vascular density and increased pericyte coverage to varying degrees (Fig. 2a–d for LLC and Supplementary Fig. 1A–D for B16). These observations are consistent with the notion that chemotherapy induces vascular regression^20^. In line with previous results^3^, comparison of WT and Mut mice reveals that the loss of myeloid cell-derived VEGF results in lower levels of VEGF within the tumours (Fig. 2a for LLC and Supplementary Fig. 1A for B16), as well as in vascular normalization (Fig. 2b–d for LLC and Supplementary Fig. 1B–D for B16), increased pericyte coverage (Fig. 2d for LLC and Supplementary Fig. 1D for B16) and decreased tumour hypoxia (Fig. 2e,f for LLC and Supplementary Fig. 1E for B16). Although vascular normalization and improved oxygenation is associated with accelerated tumour growth in untreated Mut tumours, it paradoxically results in delayed tumour outgrowth after chemotherapy (Fig. 1d,f for LLC and Fig. 1e,g for B16). The apparent contradiction arises from the augmented delivery of chemotherapeutic agent to the tumour, indicated by increased levels of cisplatin–DNA adducts (Fig. 2g,h for LLC and Supplementary Fig. 1F B16). Thus, normalizing the vasculature by targeting VEGF-A exerts opposing effects on tumour growth kinetics pre- and post chemotherapy.

### Therapy-induced senescence promotes immune cell recruitment

**Figure 3:**
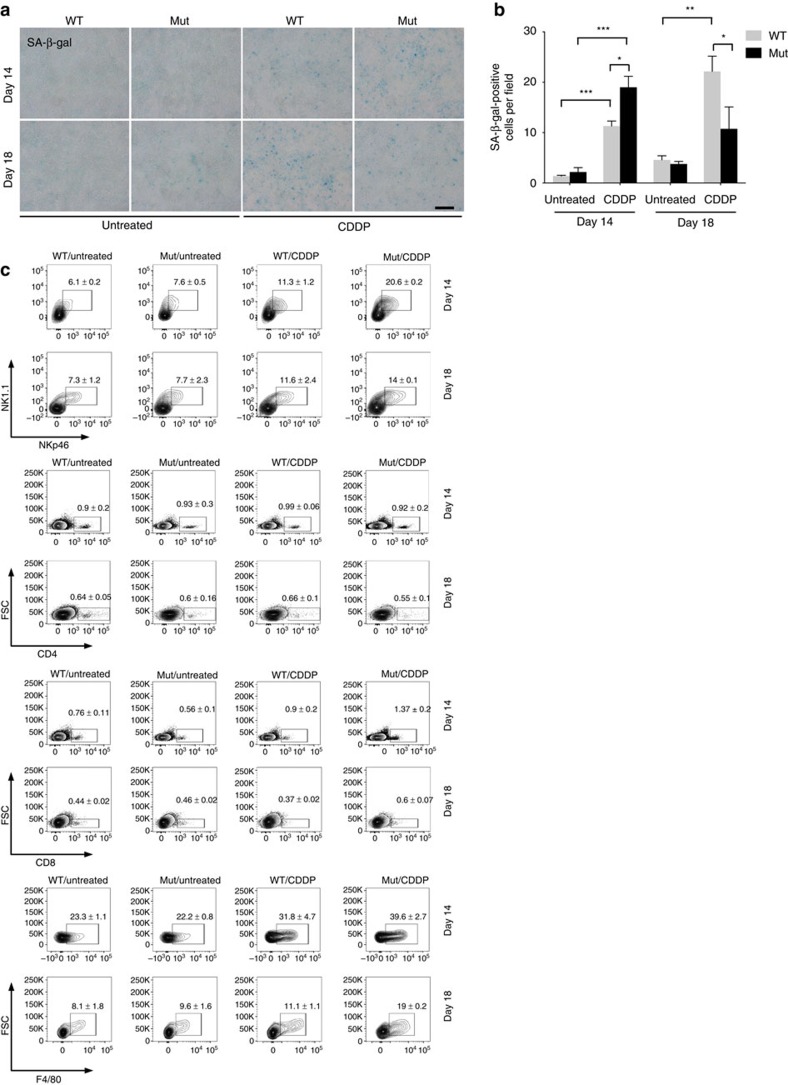
Targeting myeloid cell VEGF-A enhances chemotherapy-induced senescence and immune cell recruitment. (**a**) Representative images of untreated and CDDP-treated tumour sections showing SA-β-gal-activity at pH 6.0 at indicated time points. (**b**) Quantification of SA-β-gal-positive cells shown in **a** (untreated, *n*≥4; CDDP, *n*≥6). (**c**) Flow cytometric analysis of cisplatin-treated LLC tumours showing percentages of tumour-infiltrating NKp46/NK1.1-, CD4-, CD8- and F4/80-positive cells among CD45 cells at indicated time points (representative of three independent experiments). The full gating strategy is shown in Supplementary Fig. 9. Bars represent mean values; error bars indicate the s.e.m.; statistical significance was determined by one-way analysis of variance followed by Bonferroni *post-hoc* test when more than two groups were compared. Statistical significance is indicated as *P<0.05, **P<0.01 and ***P<0.001. Scale bar, 100 μm.

We have previously reported increased tumour cell apoptosis after chemotherapy of tumour-bearing Mut mice^3^. Not only do chemotherapeutic agents drive the apoptosis of tumour cells, they may be associated with a wide range of other outcomes including so-called premature or therapy-induced senescence^21^,^22^. Untreated tumours show hardly any senescence irrespective of the genotype (Fig. 3a,b, untreated for LLC and Supplementary Fig. 2A for B16 untreated). Deleting VEGF-A in myeloid cells increases tumour cell death on chemotherapy^3^ and promotes therapy-induced senescence, as shown by senescence-associated β-galactosidase (SA β-Gal) staining (Fig. 3a,b, day 14, CDDP for LLC and Supplementary Fig. 2A, day 16, CDDP for B16). The changes presumably stem at least partially from improved drug delivery. Tumours from Mut mice show a decline in SA β-Gal-positive cells at the endpoint (day 18 for LLC and day 20 for B16), whereas the number of senescent cells increases further in WT tumours (Fig. 3a,b, day 18, CDDP for LLC and Supplementary Fig. 2A, day 20, CDDP for B16). It thus seems likely to be that targeting VEGF-A in myeloid cells improves the clearance of senescent cells after chemotherapy.

Senescent cells remain viable and secrete a range of inflammatory cytokines^23^. The senescence-associated secretory phenotype is believed to trigger an immune response involving macrophages and NK cells that facilitates immune cell-mediated tumour clearance^24^,^25^,^26^. We used flow cytometry to analyse tumour infiltration by immune cells following chemotherapy. There were markedly higher numbers of NK cells (NKp46) and macrophages (F4/80) in the absence of myeloid cell-derived VEGF (Fig. 3c for LLC and Supplementary Fig. 2B for B16), whereas numbers of intratumoural T-helper cells (CD4) and cytotoxic T cells (CD8) were unaffected or only slightly altered (Fig. 3c for LLC and Supplementary Fig. 2B for B16). Cisplatin treatment caused transient increases in the expression of particular senescence-associated secretory phenotype cytokines (IL-6, Ccl2 and Vcam1) in tumours in Mut mice (day 14) (Supplementary Fig. 3C for LLC and Supplementary Fig. 2C for B16) and cannot explain the enhanced recruitment of immune cells after chemotherapy in Mut mice (Fig. 3c,d and Supplementary Figs 2B and 3A).

### Chemerin is released by ECs on chemotherapy

**Figure 4:**
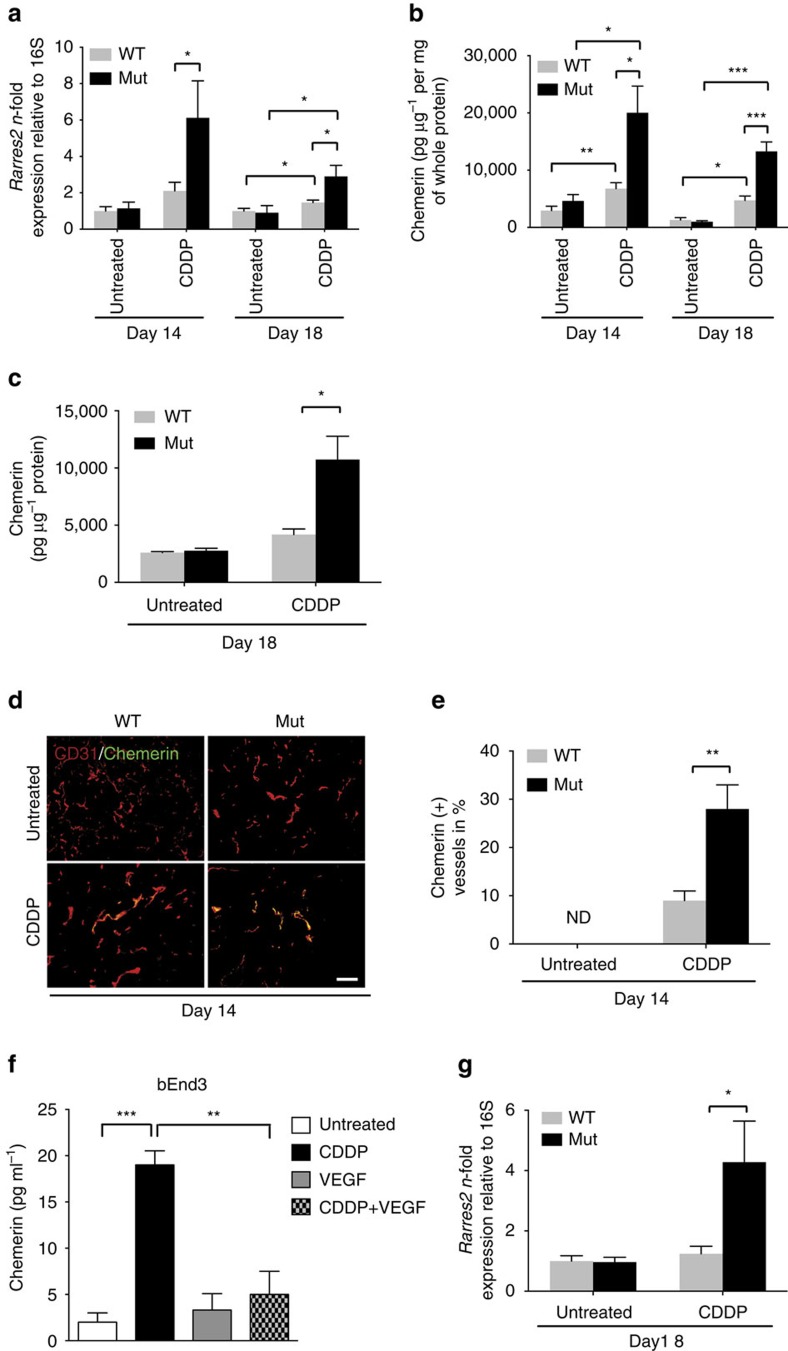
Inactivation of VEGF-A in myeloid cells enhances endothelial expression of chemerin on chemotherapy. (**a**) Quantification of *RARRES2* gene expression by quantitative real-time analysis in LLC tumours at indicated time points (untreated: *n*≥4; CDDP: *n*≥11). (**b**) Determination of levels of chemerin protein in LLC tumours on day 14 and 18 (*n*≥4). (**c**) Serum levels of chemerin in LLC-bearing mice at day 18 (untreated: *n*=4; CDDP, *n*≥8). (**d**) Representative immunofluorescent staining for CD31 and chemerin in LLC tumours. Arrows indicate positive vessels. Scale bar, 100 μm. (**e**) Quantification of chemerin-positive blood vessels as percentage of CD31-positive vessels in LLC tumours (*n*≥6). (**f**) Quantification of levels of chemerin protein of *in vitro*-cultured bEnd3 cells treated with cisplatin (3 μg ml^−1^) alone or with addition of murine recombinant VEGF (25 ng ml^−1^) for 24 h. Untreated cells served as control (*n*=3). (**g**) *N*-fold change in chemerin expression of ECs isolated from LLC tumours at day 18 (untreated, *n*≥4; CDDP, *n*=7). Bars represent mean values; error bars indicate the s.e.m.; statistical significance was determined by one-way analysis of variance followed by Bonferroni *post-hoc* test when more than two groups were compared. Statistical significance is indicated as *P<0.05, **P<0.01 and ***P<0.001. Scale bar, 100 μm.

A number of cytokines and chemokines are involved in the recruitment of immune cells to malignant tumours^27^,^28^. The chemoattractant chemerin has a crucial role in immune cell trafficking^14^,^15^,^16^. Forced expression of chemerin in tumour cells gives rise to an NK cell-based antitumour response and restricts tumour growth, whereas low levels of chemerin are associated with tumour progression and a poor outcome^19^. Increasing the level of chemerin within the tumour may thus represent a promising therapeutic approach. We found that chemotherapy increases the level of chemerin in tumours in WT mice (Fig. 4a,b for LLC and Supplementary Fig. 4A,B for B16). Surprisingly, the effect was significantly enhanced in Mut mice, showing that the absence of VEGF-A in myeloid cells stimulates the chemotherapy-evoked expression of chemerin (Fig. 4a,b and Supplementary Fig. 4A,B) and, in the LLC but not B16 model, drastically increases the levels of circulating chemerin (Fig. 4c for LLC and Supplementary Fig. 4C). Of note, chemerin messenger RNA levels are more than tenfold higher in endothelial cells (ECs) isolated from cisplatin-treated Mut LLC tumours compared with ECs isolated from cisplatin-treated Mut B16 tumours (Supplementary Fig. 4E). This suggests that only systemically elevated chemerin levels confer protection against chemotherapy-induced cachexia.

So far, the physiological source of chemerin within the tumour microenvironment has not been determined. The tumour cells themselves do not release chemerin as a consequence of chemotherapy: treatment of LLC cells *in vitro* with 3 μg ml^−1^ cisplatin, a concentration that causes a significant DNA damage response (Supplementary Fig. 5A), did not trigger chemerin release (Supplementary Fig. 5B). Similarly, cisplatin treatment of B16F10 cells produced no increase in the basal level of chemerin secreted (Supplementary Fig. 5B). Consistently, immunohistochemical analysis of tumour sections revealed only subtle chemerin reactivity in untreated LLC tumours of WT and Mut mice, as well as in tumours from cisplatin-treated WT animals (Fig. 4d). However, tumours from Mut mice showed significant chemerin immunoreactivity of the tumour vasculature on chemotherapy (Fig. 4d,e). The result indicates that tumour ECs release chemerin in response to chemotherapy, and that VEGF-A from myeloid cells suppresses the release.

To test this hypothesis, we analysed the release of chemerin by the murine EC line bEnd3. Cisplatin treatment (3 μg ml^−1^) (Fig. 4f) caused a pronounced induction of chemerin release, accompanied by the accumulation of the transcription factor peroxisome proliferator-activated receptor-γ (PPAR-γ) (Supplementary Fig. 5C,D), which stimulates chemerin expression^29^. The addition of exogenous murine VEGF-A suppresses the effect (Supplementary Fig. 5C,D) and blocks the increased production of chemerin (Fig. 4f). Comparable results were obtained in ECs isolated from tumours of both genotypes. Chemerin and PPAR-γ showed increased expression only in ECs of tumours derived from Mut mice after chemotherapy (Fig. 4g and Supplementary Fig. 5E for LLC and Supplementary Figs 5F and 4D for B16), confirming that ablation of myeloid cell-derived VEGF-A significantly increases the expression of chemerin in response to chemotherapy. Interestingly, exogenous addition of another angiogenic factor, basic fibroblast growth factor also inhibited cisplatin-induced chemerin release from bEnd3 cells (Supplementary Fig. 5G).

Consistently, chemotherapy with another frequently used cytotoxic agent, etoposide, resulted in improved chemotherapeutic outcome in LLC-bearing Mut mice, increased chemerin expression in tumour ECs, enhanced NK cell recruitment and decreased senescent tumour cells (Supplementary Fig. 6A–D, respectively) but failed to increase systemic chemerin levels (Supplementary Fig. 6E), further supporting a link between systemic chemerin levels and protection against cachexia. It is noteworthy that in contrast to cisplatin, etoposide treatment caused only a very mild exacerbation of cachexia without genotype-specific differences (Supplementary Fig. 6F).

Interestingly, treatment of LLC-bearing mice with a VEGF-neutralizing antibody at a dose that induced vascular changes reminiscent of vascular normalization (Supplementary Fig. 7A,B) improved treatment outcome on chemotherapy with cisplatin (Supplementary Fig. 7C) endothelial chemerin expression and NK cell recruitment (Supplementary Fig. 7D,E). Importantly, anti-VEGF treatment under these circumstances had no impact on levels of circulating chemerin or cisplatin-exacerbated cachexia (Supplementary Fig. 7F,G). The data verify the tumour endothelium as a source of chemerin in response to chemotherapy and show that chemerin production is suppressed by VEGF-A.

### Chemerin prevents therapy-associated loss of body weight

**Figure 5:**
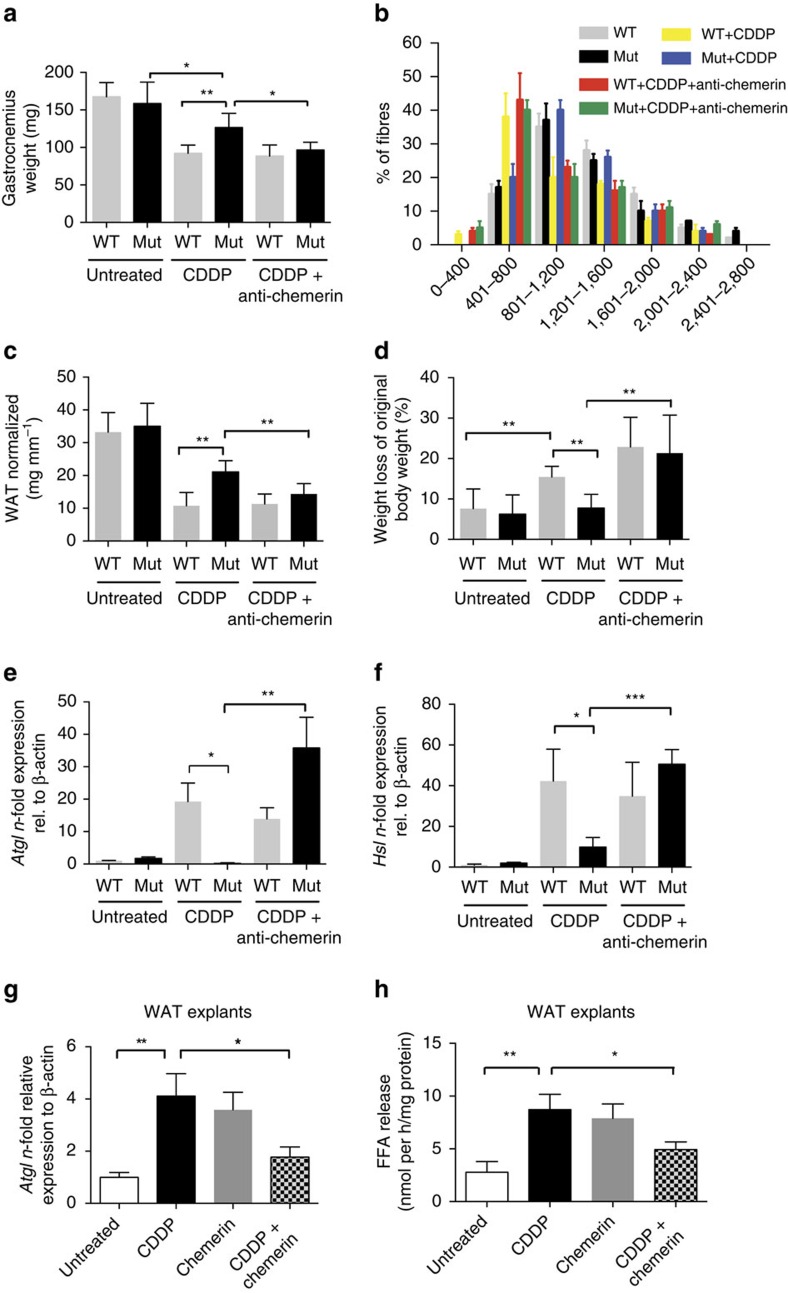
Chemerin protects Mut (LysMCre/VEGF^f^/^f^) mice from chemotherapy-induced lipolysis and skeletal muscle loss. (**a**) Weight of gastrocnemius muscle in LLC tumour-bearing mice without treatment and after administration of CDDP alone or with chemerin-neutralizing antibody on day 18 (WT: n *n*≥4; Mut: *n*≥7). (**b**) The cross-sectional area of gastrocnemius muscle fibres from LLC tumour-bearing mice are represented as a frequency histogram from *n*=2 mice. The mean cross-sectional area of the fibres in μm^2^ is indicated on the *x* axis. (**c**) Amount of WAT normalized to tibia length of untreated, cisplatin-treated and cisplatin+anti-chemerin-treated LLC-bearing mice on day 18 (WT: *n*≥4; Mut: *n*≥7). (**d**) Weight loss of LLC tumour-bearing mice on day 18. Weight loss is given as percentage of the original body weight (WT: *n*≥4; Mut: *n*≥7). (**e**) *N*-fold change in gene expression of *Atgl* in gonadal fat tissue from LLC-bearing mice on day 18 (WT: *n*≥4; Mut: *n*≥7). (**f**) *N*-fold change in gene expression of *Hsl* in gonadal fat tissue from from LLC-bearing mice on day 18 (WT: *n*≥4; Mut: *n*≥7). (**g**) Quantification of levels of *Atgl* transcripts in explant cultures of WAT from C57/Bl6J-WT mice. Explants were treated for 24 h as indicated (*n*≥4). (**h**) Colorimetric determination of free fatty acid (FFA) release in supernatants from WAT explants treated as described in **g** (*n*≥4). Bars represent mean values; error bars indicate the s.e.m.; statistical significance was determined by one-way analysis of variance followed by Bonferroni *post-hoc* test when more than two groups were compared. Statistical significance is indicated as *P<0.05, **P<0.01 and ***P<0.001.

Chemerin was initially described as an adipokine with context-dependent pro- and antilipolytic effects on WAT^13^,^30^, as well as a regulator of skeletal muscle homeostasis^31^,^32^. It seemed conceivable that changes in chemerin levels could affect lipid and muscle metabolism in our LLC-bearing Mut mice, which were protected against chemotherapy-exacerbated chemotherapy. It was initially important to assess the contribution of loss of adipose tissue and skeletal muscle to the overall weight loss associated with chemotherapy. We thus weighed gastrocnemius muscles and gonadal adipose depots in LLC-bearing mice subjected to chemotherapy. Consistent with the concept that cachexia involves breakdown of skeletal muscle and WAT, chemotherapy of WT mice resulted in a >30% loss of gastrocnemius weight along with a reduction in muscle fibre size (Fig. 5a,b) and in a >60% reduction in gonadal WAT (Fig. 5c) along with overall loss of body weight (Fig. 5d). The expression of the major lipolytic enzymes Atgl and Hsl in WAT isolated from cisplatin-treated WT mice was significantly upregulated (Fig. 5e,f). The increase in lipolytic enzymes and muscle degradation depended on the presence of myeloid-derived VEGF-A: chemotherapy of Mut mice caused a far smaller loss of gastrocnemius weight and WAT (Fig. 5a,c).

The data suggest that differences in chemerin release underlie not only the altered tumour immune cell infiltration but also the striking difference in weight loss between WT and Mut mice following chemotherapy. To test this interpretation, we depleted chemerin by means of an anti-chemerin antibody. Remarkably, the antibody caused Mut mice to suffer the same loss of body weight (Fig. 5d), skeletal muscle (Fig. 5a,b) and WAT (Fig. 5c) as WT mice on cisplatin treatment. Furthermore, following chemotherapy the *Atgl* and *Hsl* genes were expressed at similar levels in WT mice and in Mut mice treated with the antibody (Fig. 5e,f). The differences in weight and WAT loss on chemotherapy could not be accounted for by variations in food intake, which did not depend on genotype, although chemotherapy resulted in a reduced food intake in both WT and Mut mice (Supplementary Fig. 8A). Likewise, serum levels of the cachexia-inducing cytokines TNF-α and IL-6 were similar across genotypes and treatment regimens (Supplementary Fig. 8B). The protection from chemotherapy-induced cachexia in Mut mice is thus associated with the loss of myeloid cell-derived VEGF-A and the resulting increase in the level of circulating chemerin.

The cause of weight loss associated with chemotherapy is poorly understood. Our findings suggest that in addition to a proteolytic effect on skeletal muscle, cisplatin might have a strong and direct lipolytic effect that is modulated by chemerin. To investigate the possibility, gonadal WAT explants from C57Bl6/J mice were treated with cisplatin, which was found to induce *Atgl* expression (Fig. 5g) and to stimulate release of fatty acids (Fig. 5h). The expression of *Hsl* was also induced, although not significantly (Supplementary Fig. 8C). Consistent with its context-specific role in enhancing or inhibiting lipolysis, chemerin increased *Atgl* expression and lipolysis in WAT explants (Fig. 5g,h) but suppressed the increased expression of *Atgl* and WAT lipolysis caused by addition of cisplatin (Fig. 5g,h). The experiment confirms that cisplatin directly stimulates WAT lipolysis, and that the effect is negated by chemerin, which thereby protects against therapy-associated loss of body weight.

### Local and systemic effects of chemerin amend therapy outcome

**Figure 6:**
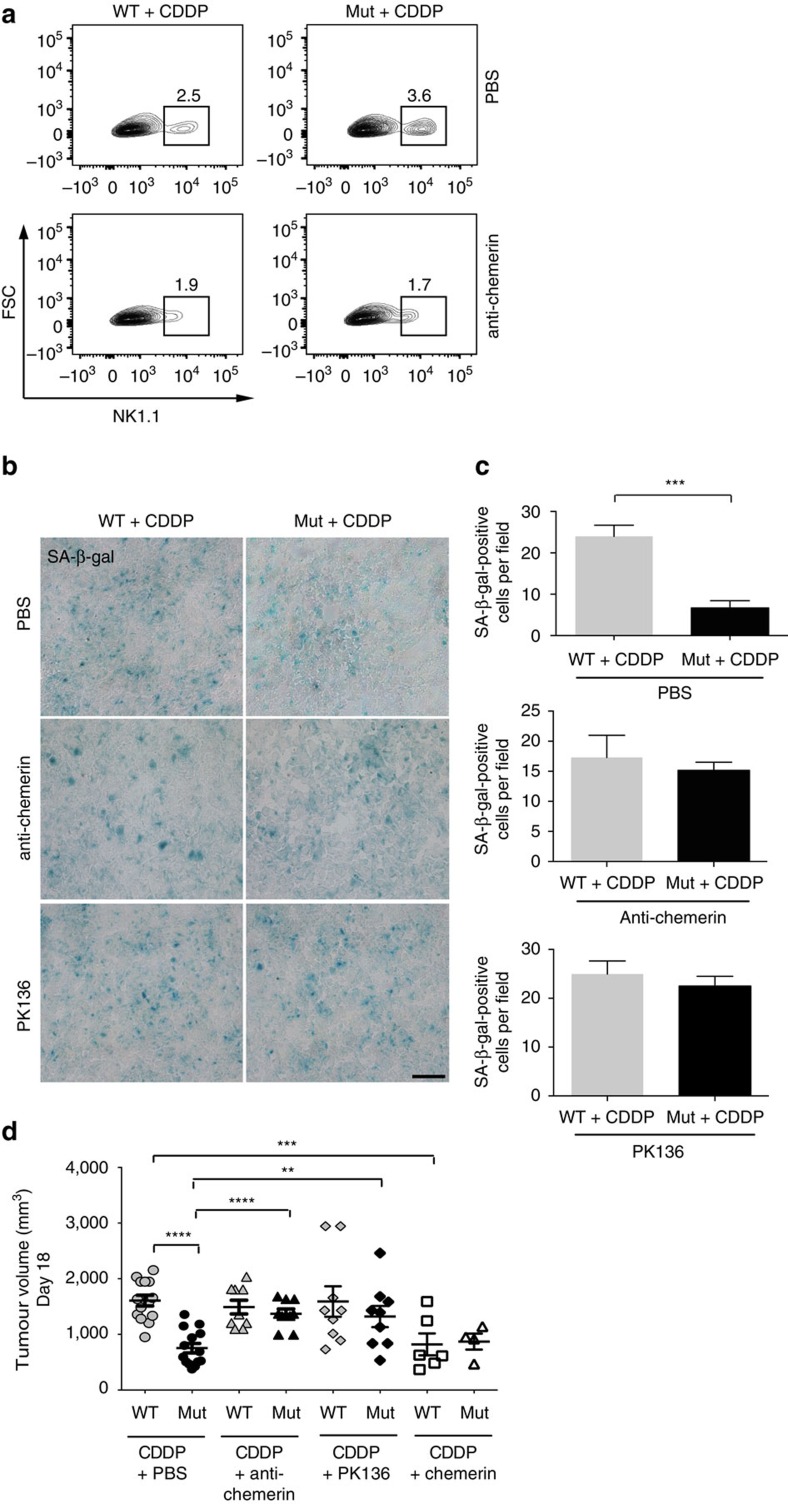
Chemerin release and NK cell anti-tumour defense account for the improved growth restriction on cisplatin treatment in Mut (LysMCre/VEGF^f^/^f^) mice. (**a**) Percentage of NK1.1-positive cells in cisplatin-treated tumours from WT and Mut mice that received i.p. injections of PBS as control (upper panel) or chemerin-neutralizing antibody (lower panel), determined by flow cytometry. Tumours were analysed in three independent experiments. (**b**) Representative micrographs of SA-β-gal-activity at pH 6.0 on day 18 in tumour sections after treatment with CDDP alone (upper panel), with CDDP and chemerin-neutralizing antibody (middle panel) or with CDDP and NK-depleting antibody mAB PK136 (lower panel). (**c**) Quantification of SA-β-gal-positive cells in **b** (untreated, *n*≥4; CDDP, *n*≥6). (**d**) Tumour volumes on day 18 after treatment with CDDP alone, CDDP and chemerin-neutralizing antibody, CDDP and mAB PK136 or CDDP and intratumoural injections of recombinant chemerin (*n*≥5). Bars represent mean values; error bars indicate the s.e.m.; statistical significance was determined by one-way analysis of variance followed by Bonferroni *post-hoc* test when more than two groups were compared. Statistical significance is indicated as *P<0.05, **P<0.01 and ***P<0.001. Scale bar, 100 μm.

**Figure 7:**
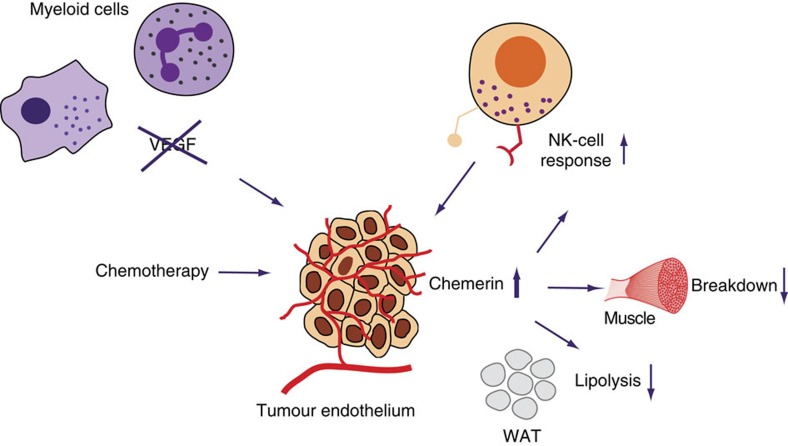
Graphical summary and proposed mechanism. Proposed model for the improved outcome of chemotherapy and prevention of body weight loss by targeting VEGF in myeloid cells: increased levels of circulating chemerin due to enhanced release of chemerin by the tumour endothelium improve NK cell recruitment to the tumour and prevent skeletal muscle loss and WAT lipolysis.

Chemotherapy causes an increase in the intratumoural release of chemerin in Mut mice. Chemerin might thus be involved in the enhanced immune response in the absence of myeloid cell-derived VEGF-A, which is associated with the improved control of tumour growth. The interpretation was tested by means of an anti-chemerin antibody, which diminished chemotherapy-induced recruitment of NK cells in WT and Mut mice (Fig. 6a). The antibody completely blocked the clearance of senescent tumour cells after cytotoxic treatment in the absence of myeloid cell-derived VEGF-A, resulting in equal numbers of senescent cells in tumours from WT and Mut mice at endpoint (Fig. 6b,c). Blocking chemerin led to comparable outcomes in WT and Mut mice at endpoint (Fig. 6d). Comparable results were obtained by depleting NK cells (Fig. 6d). In the absence of NK cells, senescent cells were not cleared and remained in Mut tumours on regrowth (Fig. 6b,c) and there was no delay in tumour growth after chemotherapy (Fig. 6d). Finally, intratumoural injection of chemerin delayed tumour regrowth after chemotherapy in WT mice but had no additional effect in Mut mice (Fig. 6d). However, intratumoural chemerin injection does not further affect circulating chemerin levels in tumour-bearing and cisplatin-treated WT and Mut mice (Supplementary Fig. 8D). Moreover, neither local application of chemerin nor NK cell depletion protected against chemotherapy-induced weight loss (Supplementary Fig. 8E). Therefore, local and systemic chemerin effects need to be distinguished. The findings unequivocally link the improved outcome of chemotherapy in the absence of myeloid cell-derived VEGF-A to sufficient release of the chemoattractant chemerin by the endothelium, which locally activates NK cell-based antitumour defenses and prevents chemotherapy-exacerbated cachexia at the systemic level (graphical summary, Fig. 7).

## Discussion

Targeting VEGF-A in myeloid cells leads to vascular normalization^3^. Here we show that targeting VEGF-A is also associated with an enhanced senescence response on chemotherapy. In addition to improved drug delivery, the reduced tumour hypoxia in Mut tumours may contribute to the effect, as hypoxia has been reported to prevent cellular senescence^33^. Although T-cell-mediated immune responses are impaired by a lack of oxygen^34^, it remains to be determined how NK cells react under hypoxic conditions. It is attractive to speculate that the reduced hypoxia in Mut mice improves NK cell-mediated cytotoxicity.

In addition to shaping the tumour vasculature, VEGF-A modulates the performance of various immune cells^35^. It may have an effect on the migration and cytotoxicity of NK cells, although findings are inconsistent^36^,^37^. It clearly attracts regulatory T cells to the tumour microenvironment^38^ and interferes with the maturation of dendritic cells^35^. The absence of myeloid cell-derived VEGF-A from the tumour microenvironment could thus improve antitumour immune responses.

The chemotherapeutic agent cisplatin reduces vascular density and increases pericyte coverage, consistent with its known anti-angiogenic properties^20^. The effect is independent of myeloid cell-derived VEGF-A, although the density of blood vessels before chemotherapy is higher in tumours from WT mice than in those from mutant mice lacking VEGF-A in myeloid cells. The reduction in tumour blood vessels on chemotherapy may thus be enhanced by VEGF-A. The effect may stem from improved drug delivery and/or be related to the presumably higher number of proliferating ECs on VEGF-A-driven angiogenesis. The proliferating cells in the vasculature would be more susceptible to cytotoxic damage than quiescent cells.

Our study reveals that chemotherapy increases the level of PPAR-γ within tumour ECs and stimulates them to release chemerin. However, only in the LLC model deletion of VEGF in myeloid cells resulted in increased systemic chemerin levels, whereas in the B16 model only local, intratumoural effects were observed. Local and systemic chemerin effects need to be distinguished. It is attractive to speculate that only sufficently elevated systemic (circulating) chemerin levels are able to ameliorate cisplatin-induced cachexia. These systemic and therefore cachexia-relevant effects need to be distinguished from local, intratumoural effects of chemerin, for example, clearance of senescent tumour cells and restriction of tumour growth. Therefore, local delivery by intratumoural injection of chemerin phenocopies (local) reduction of tumour size (Fig. 6d) but fails to induce systemic effects (Supplementary Fig. 8E) in LLC-bearing cisplatin-treated WT mice. Consistent with this hypothesis, intratumoural chemerin injection does not further affect circulating chemerin levels in tumour-bearing and cisplatin-treated WT and Mut mice (Supplementary Fig. 8D). Likewise, deletion of VEGF in myeloid cells does not confer protection against cisplatin-induced cachexia in the B16 model (Fig. 1i). Again, the differences are in local versus systemic effects. This might again be due to the lack of increased circulating chemerin levels in cisplatin-treated Mut mice in the B16 model (Supplementary Fig. 4C) compared with the LLC model (Fig. 4c). With the aim to reconcile the contradictory results we compared absolute chemerin mRNA expression levels in addition to *n*-fold expression as in the study, in isolated ECs, which we have identified as the major source of chemerin (Fig. 4d–g) from LLC and B16 tumours across genotypes. As shown in Supplementary Fig. 4E, chemerin mRNA levels are more than tenfold higher in ECs isolated from cisplatin-treated Mut LLC tumours compared with ECs isolated from cisplatin-treated Mut B16 tumours. In line with this, in the B16 model serum chemerin levels of cisplatin-treated Mut mice are lower than in the LLC model (Fig. 4c and Supplementary Fig. 4C, respectively). This could explain why increased circulating chemerin levels and therefore systemic protection against chemotherapy-induced cachexia are only achieved in cisplatin-treated Mut LLC tumours, whereas local, intratumoural effects are observed in all models. Currently, we can only speculate regarding the different chemerin levels between tumour models. One reason might be that the tumour VEGF levels after cisplatin treatment in B16 tumours are generally higher (Supplementary Fig. 1A) than in LLC tumours (Fig. 2a) and, therefore, endothelial chemerin release is still repressed in B16 tumours. Alternatively, the increased expression of other angiogenic factors (for example, fibroblast growth factor; Supplementary Fig. 5G) in the B16 model may repress endothelial chemerin expression in cisplatin-treated Mut mice (Supplementary Fig. 1E). Consistently, only increased serum levels in LLC-bearing Mut mice conferred protection against chemotherapy-induced cachexia.

The role of chemerin in skeletal muscle homeostasis is controversial^31^,^32^ and the effect of chemerin on muscle loss in the context of cachexia is unknown. Our *in vivo* experiments show that chemerin prevents excessive loss of skeletal muscle on chemotherapy. Likewise, chemerin has opposing effects on lipid metabolism depending on the nutritional status and on other factors. *In vitro* experiments show that chemerin may have pro- or antilipolytic effects depending on the experimental conditions^13^,^30^. *In vivo* evidence is limited, although treatment of fasted mice with chemerin is known to inhibit lipolysis and release of free fatty acids^30^. Consistently, we show that lipolysis and the release of free fatty acids are downregulated by the addition of chemerin to WAT cultures after the chemotherapeutic induction of lipolysis. In contrast, chemerin treatment of WAT explants before chemotherapy induces lipolysis. We speculate that chemerin acts as a rheostat in the homeostasis of fat tissue, preventing excessive accumulation or depletion of fat reserves in the presence of powerful anti- or prolipolytic stimuli.

Tumour ECs release chemerin in response to chemotherapy but the effect is suppressed by VEGF-A derived from myeloid cells. Lowering intratumoural levels of VEGF-A after chemotherapy thus has an additional important effect: as well as normalizing the vasculature, it also fosters the endothelial production of chemerin. Consistently, elimination of myeloid cell-derived VEGF-A has a similar local effect (for example, tumour size restriction and increased NK cell infiltration as shown in Supplementary Fig. 6A–D) when etoposide, another cytotoxic agent, is used. Regarding the results in etoposide-treated LLC tumours, we would like to emphasize that etoposide treatment at the indicated dose phenocopies the intratumoural and hence local effects of cisplatin treatment in LLC-bearing Mut mice (Supplementary Fig. 6A–D) and fails to increase systemic chemerin levels (Supplementary Fig. 6E). Furthermore, etoposide at this dose induces only very mild cachexia (Supplementary Fig. 6F) compared with cisplatin treatment (Fig. 1h,i), although it still slows tumour growth (Supplementary Fig. 6A). Therefore, in this setting of overall weak chemotherapy-induced cachexia, potential protective effects against chemotherapy-induced cachexia by targeting myeloid cell–VEGF might not become apparent. Moreover, cisplatin and etoposide are non-immunogenic^39^ and it will be important to investigate the effects on chemerin release of other immunogenic chemotherapeutics.

It is noteworthy that treatment with a VEGF-neutralizing antibody induced vascular normalization, improved the outcome of chemotherapy, endothelial chemerin expression and NK cell recruitment. Yet, anti-VEGF treatment under these particular conditions had no effect on cisplatin-exacerbated cachexia, presumably owing to the inability to increase systemic chemerin levels. Myeloid cell-derived VEGF has indeed been shown to play a unique role in VEGFR2-mediated signalling to the tumour endothelium that cannot be compensated for by other potential VEGF sources within the tumour microenvironment (for example, tumour cells), regardless of overall tumour VEGF levels^3^. This is attributed to the ability of myeloid cells (in particular macrophages) to generate transiently and locally very high VEGF concentrations in restricted tumour areas, which is not necessarily reflected by total VEGF levels within the tumour. Moreover, the mostly perivascular localization of tumour-associated macrophages puts them in a unique position and makes them presumably a critical and non-redundant source of VEGF directly adjacent to the abluminal side of the endothelium. This may explain why antibody-mediated general VEGF neutralization, predominantly targeting circulating VEGF, is less efficient than genetic targeting of VEGF in myeloid cells, in particular with regard to increasing endothelial chemerin release and systemic levels that are relevant for the protection against cachexia. However, general VEGF blockade in combination with cisplatin is still able to phenocopy the local effects, restricted to the tumour microenvironment (for example, tumour growth inhibition, vascular phenotype and immune cell infiltration) (Supplementary Fig. 7).

The tumouricidal effects of many chemotherapeutic agents depends on the active contribution of immune cell effectors, especially those of the adaptive immune compartment^1^. In our tumour models, therapeutic success critically depends on NK cell-mediated tumour immune surveillance and tumour cell clearance. It is important to note that not all tumours are sensitive to NK cell-mediated tumour surveillance. Further work will be necessary to evaluate the outcome of drug-induced senescence and stromal chemerin release in tumour models that are predominantly controlled by T cells.

In summary, our study reveals that chemotherapy with cisplatin simulates tumour ECs to release chemerin. We show further that chemerin is a critical mediator of NK cell-mediated antitumour defenses and of cachexia as well (Scheme, Fig. 7). VEGF-A derived from myeloid cells suppresses the stimulation of endothelial chemerin release by chemotherapy. Hence, targeting VEGF signalling should impede the lipolysis and weight loss that is frequently associated with chemotherapy. Our study therefore offers novel therapeutic avenues to improve the overall outcome of chemotherapy.

## Methods

### Animals and procedures

The Animal Care and Use Committee of the Bezirksregierung Düsseldorf, Germany, approved all procedures performed on mice. Mice (C57Bl/6J) with both alleles of exon 3 of VEGF-A flanked by loxP sites (VEGF+^f^/+^f^)^40^ were bred with mice (C57Bl/6J) homozygous for the floxed VEGF allele expressing Cre recombinase driven by the lysozyme M promoter^41^ (LysMCre+/VEGF+^f^/+^f^)^3^. Male mice at 10–12 weeks of age (C57Bl/6J) were used. Chemotherapy was started 8 days after subcutanous injection of 10^7^ LLC cells and 10 days after injection of 10^7^ B16F10 cells. Three doses of cisplatin (8 mg per kg body weight, Sigma) or etoposide (15 mg kg^−1^, Sigma) were given by intraperitoneal (i.p.) injection every 2 days. Tumour size was monitored every 2 days using a caliper and the tumour volume was calculated as V=*π*/6*A × B2. Tumours were allowed to grow until the maximum permitted size was reached or ulcerations occured. Pimonidazole hydrochloride (Hypoxyprobe-1) was injected i.p. (60 mg per kg body weight) 30 min before tumour removal and detected by the monoclonal antibody Mab-1. Tumour doubling time was calculated as DT=(*T*−T0) × ln2/(lnV−lnV0), where T−T0 indicates the time between two measurements and V0 and V denote the tumour volume at these times. Mice of different genotypes were allocated randomly to the different treatment groups and analysis was carried out in a single-blinded manner. Sample size was estimated based on previous studies with the experimental models^3^.

### Depletion of NK cells

After the last cytotoxic treatment, randomized cohorts of WT and Mut mice were injected i.p. with anti-NK1.1 monoclonal antibody PK136 (4 mg per kg body weight) at day 13 and 15. Control mice received i.p. injections of 100 μl PBS.

### Chemerin neutralization

Randomized cohorts of WT and Mut mice received i.p. injections of 400 μg per kg body weight anti-chemerin (R&D Systems) on days 11, 13 and 15. Control mice were injected i.p. with PBS.

### Assessment of skeletal muscle

Gastrocnemius muscles were dissected and used to assess muscle atrophy. These muscles were frozen and 7 μm-thick serial sections were stained histochemically for myofibrillar ATPases. Morphometric analyses were then performed on these muscles to determine the cross-sectional area of fibres for each group; *n*=2 mice for the gastrocnemius muscle.

### Chemerin injection

Randomized cohorts of WT and Mut mice received intratumoural injections of 250 ng of recombinant, active, carrier-free murine chemerin (R&D Systems) reconstitued in PBS daily starting on day 6 until endpoint at day 18. Control mice were injected with PBS.

### VEGF neutralization

WT mice received three i.p. injections at days 7, 9 and 11 of 100 μg of rat anti-mouse VEGF-A antibody (Biolegend, #512808). Control mice were injected with isotype control (#400533).

### Primary antibodies

Rat anti-CD31 (BD Biosciences, #553370) at 1:200 and rat anti-CD31 (Dianova, #DIA-310) at 1:100, mouse anti-SMA-α (Chemicon, #CBL171) at 1:200, rat anti-Ki-67 (Abcam, #ab15580) at 1:500, goat anti-chemerin (R&D Systems, #AF2325) at 1:100, mouse anti-MAB-1 (Hypoxyprobe-1, #Mouse-Mab1) at 1:100, rabbit anti-PPAR-γ (Abcam, ab#59256) at 1:500, rabbit anti-β-actin (Santa Cruz Biotechnology, #sc-47778) at 1:1,000 and rabbit anti-NKI-A59 (a gift from B. Floot, Netherlands Cancer Institute, Amsterdam, Netherlands) at 1:100 were used as primary antibodies.

### RNA extraction and reverse transcriptase–quantitative PCR

Total RNA was isolated by phenol/chloroform extraction. Complementary DNA was synthesized from 3 μg of DNA-free total RNA using M-MLV Reverse Transcriptase (Promega) and oligo-dT primers (Life Technologies). Gene-specific transcription levels were determined using SYBR Green Mastermix (Promega) and an IQ5 real-time PCR machine (Bio-Rad). PCR conditions were as follows: 95 °C for 10 min followed by 40 cycles of 95 °C for 15 s and 60 °C for 1 min. Data were normalized to 16S or β-actin mRNA levels. The following primers were used: 16s forward primer: 5′-AGATGATCGAGCCGCGC-3′, reverse primer: 5′-GCTACCAGGGCCTTTGAGATGGA-3′; *β-actin* forward primer: 5′-ATGGAGGGGAATACAGCCC-3′, reverse primer: 5′-TTCTTTGCAGCTCCTTCGTT-3′; *Atgl* forward primer: 5′-GTTGAAGGAGGGATGCAGAG-3′, reverse primer: 5′-GCCACTCACATCTACGGAGC-3′; *Ccl2* forward primer: 5′-TCTCCAGCCTACTCATTGGG-3′, reverse primer: 5′-AGGTCCCTGTCATGCTTCTG-3′; *Csf-1* forward primer: 5′-TAGTGGTAGGCCACATTCCC-3′, reverse primer: 5′-GGATGAGGACAGACAGGTGG-3′; *Cxcl-1* forward primer: 5′-GTGCCATCAGAGCAGTCTGT-3′, reverse primer: 5′-GCACCCAAACCGAAGTCATA-3′; *Cxcl-2* forward primer: 5′-CATCAGGTACGATCCAGGCT-3′, reverse primer: 5′-CCTGGTTCAGAAAATCATCCA-3′; *Hsl* forward primer: 5′-CCTGTCTCGTTGCGTTTGTA-3′, reverse primer: 5′-ACGCTACACAAAGGCTGCTT-3′; *IL-1α* forward primer: 5′-TGAGTTTTGGTGTTTCTGGC-3′, reverse primer: 5′-ATGTATGCCTACTCGTCGGG-3′; *IL-1β* forward primer: 5′-TTGTTGATGTGCTGCTGTGA-3′, reverse primer: 5′-TGTGAAATGCCACCTTTTGA-3′; *IL-10* forward primer: 5′-AGACACCTTGGTCTTGGAGC-3′, reverse primer: 5′-TTTGAATTCCCTGGGTGAGA-3′; *IL-6* forward primer: 5′-TCTGAAGGACTCTGGCTTTG-3′, reverse primer: 5′-GATGGATGCTACCAAACTGGA-3′; *Ncam-1* forward primer: 5′-CTCCTTGGCTGGGAACAATA-3′, reverse primer: 5′-AAGGGGAAGGCACTGAATTT-3′; *p21* forward primer: 5′-AGAGACAACGGCACACTTTG-3′, reverse primer: 5′-CGGTGTCAGAGTCTAGGGGA-3′; *p53* forward primer: 5′-AATGTCTCCTGGCTCAGAGG-3′, reverse primer: 5′-CTAGCATTCAGGCCCTCATC-3′; *PPAR-γ* forward primer: 5′-GATGGAAGACCACTCGCATT-3′, reverse primer: 5′-AACCATTGGGTCAGCTCT-3′; *Rarres2* forward primer: 5′-TACCCTTGGGGTCCATTTTA-3′, reverse primer: 5′-TCTTCTCAGCTGGCACCTTT-3′; and *Vcam-1* forward primer: 5′-ACCAAGGAAGATGCGCAGTA-3′, reverse primer: 5′-CCGGCATATACGAGTGTGAA-3′.

### Immunofluorescence/immunohistochemistry

Optimal cutting temperature compound (OCT)-embedded frozen sections (10 μm thick) were thawed at room temperature and fixed in methanol/acetone (1:1) for 5 min at −20 °C. Sections were blocked with 5% normal goat serum (Sigma) for 60 min at room temperature and incubated with the primary antibody overnight at 4 °C. Species-specific fluorochrome-conjugated Alexa 488 and Alexa 568 (Invitrogen, #A-11034, # O-6382, # A-11055, # A-21141, #A-11011, #A-11004, #A-11077) at 1:200 were used as secondary antibodies for 30 min at room temperature. For staining with mouse-derived antibodies, Mouse on mouse (M.O.M.) Basic Kit (Vector Laboratories) was used in accordance with the manufacturer's instructions. Cell nuclei were stained with 4,6-diamidino-2-phenylindole (Invitrogen) and cover slips were mounted with Mounting Medium (Dako). The TdT-mediated dUTP nick end labelling assay was performed in accordance with the manufacturer's instructions (Promega). Chemerin/CD31 immunofluorescence was performed after heat-induced antigen retrieval.

### Senescence associated β-galactosidase staining

Ten micrometres of frozen sections of OCT-embedded tumours were stained for the senescence marker SA β-Gal according to the manufacturer's instructions (Cell Signaling) for B16F10 tumours the staining was performed with dodecanoylaminofluorescein di-β-D-galactopyranoside (C12FDG; Life Technologies) at 33 μM during 2 h at 37 °C at pH 6.0.

### Flow cytometry analysis

Tumours were digested with 0.1% collagenase type III (Worthington) and 1 U ml^−1^ DNAse I (Promega) and incubated at 37 °C for 1 h. One hundred and six tumour cells were incubated with Fc-Block (BD Biosciences, #553141 at 1/100) before labelling with fluorochrome-conjugated antibodies. The following fluorochrome-conjugated antibodies were used: anti-NK1.1 (BD Biosciences, #557391 at 1/50); anti-CD335 (NKp46, eBioscience, #25–3351 at 1/50), anti-CD45 (eBioscience, #45–0451 at 1/50), anti-CD4 (Biolegend, #100408, at 1/50), anti-CD8 (Biolegend, #100723 at 1/50) and anti-F4/80 (Biolegend, #123118 at 1/50). LIVE/DEAD Fixable Aqua Dead Cell Stain Kit (Thermo Fisher Scientific; L34957) was used as viability dye. Gating strategy was as follows: the single cell leukocyte population was selected by forward scatter (FSC)-H versus FSC-A and side scatter (SSC)-A versus FSC-A, respectively. The leukocyte population was further analysed for their uptake of the Live/Dead Aqua stain, to determine live versus dead cells and for the expression of CD45. Next, CD45+ cells were classified as NK cells by expression of NKp46 (in LLC tumours additionally with NK1.1), CD4+ T cells by CD4 expression, CD8+ T cells by CD8 expression and macrophage population by F4/80 expression. Labelled cells were analysed on a LSR II flow cytometer (BD Biosciences) and were evaluated with FlowJo Mac software, version 10.0.8r1. The gating strategy is depicted in Supplementary Fig. 9.

### Isolation of ECs

To isolate ECs, at experiment endpoint tumours were digested in cell lysis buffer (DMEM+2 mg ml^−1^ collagenase type III) for 1 h at 37 °C. Up to 4 × 10^7^ cells were incubated with mouse CD31 microbeads (Miltenyi Biotec) and ECs were separated to a purity of ≥90% by positive selection according to the manufacturer's instruction. ECs (8 × 10^5^±1,3 × 10^5^) were isolated corresponding to 5.59%±1.45% of total cells.

### Determination of free fatty acids of isolated WAT

Fat tissue explants were obtained from gonadal WAT of C57/Bl6J-WT mice and cut into small pieces. The pieces were maintained for 18 h in DMEM supplemented with 2% BSA at 37 °C and 5% CO_2_. Aliquots of medium were analysed for free fatty acids using a commercial kit (Free Fatty Acid Quantification Kit, Abcam) following the manufacturer's instructions.

### Quantitative analysis of histology markers

For quantitative analysis of blood vessels, five areas of each tumour section were randomly selected and photographed using a Nikon Eclipse E1000 microscope and the Nikon DS-Ri1 camera system. The area (number of pixels/px) marked by CD31 was measured using the ImageJ programme (National Institutes of Health) and calculated as the percentage of the area covered by 4,6-diamidino-2-phenylindole. Pericyte coverage was calculated as percentage of total number of blood vessels counted. Chemerin-positive vessels were calculated as percentage of total CD31-positive vessels. To determine cell proliferation, apoptosis and cellular senescence, cells positive for the marker in question were counted in five randomly chosen tumour areas for each section and the mean value calculated.

### ELISA assay

Concentrations of VEGF-A and chemerin in tumours and aliquots of medium were determined using commercial kits (Quantikine ELISA Immunoassay, R&D Systems) and expressed in pg ml^−1^ per mg of whole tissue protein. Serum levels of TNF-α and IL-6 were measured using mouse TNF-α and IL-6 quantikine ELISA kit (R&D Systems) and normalized to serum protein levels.

### Cell culture

Cell lines were obtained from ATCC. Cells were cultured in DMEM high-glucose medium supplemented with 10% FCS, 50 U ml^−1^ penicillin and 100 μg ml^−1^ streptomycin at 37 °C in a humidified atmosphere of 5% CO_2_ in air and were checked for *Mycoplasma* contamination.

### Western blotting

Protein samples were separated using a 10% polyacrylamide gel under reducing and denaturating conditions and transferred onto a polyvinylidene difluoride membrane followed by enhanced chemiluminescence (ECL) detection of the antibody. For quantitative analysis, the membranes were scanned with the ImageQuant LAS 4000mini (GE Healtcare Life Sciences) and the integrated density was measured using the software ImageJ (National Institutes of Health). Images have been cropped for presentation. Full-size images are presented in Supplementary Fig. 10.

### Statistical analysis

Statistical analysis was performed with the Prism 6.0 software (GraphPad Software). Statistical significance was determined by an unpaired Student's *t*-test or one-way analysis of variance, where appropriate. Statistical significance is indicated as *P<0.05, **P<0.01, ***P<0.001 and ****P<0.0001.

### Data availability

The data that support the findings of this study are available from the corresponding author upon request.

## Additional information

**How to cite this article:** Klose, R. *et al.* Targeting VEGF-A in myeloid cells enhances natural killer cell responses to chemotherapy and ameliorates cachexia. *Nat. Commun.* 7:12528 doi: 10.1038/ncomms12528 (2016).

## Supplementary Material

Supplementary Information Supplementary Figures 1-10
