# Supplementary material for: Targeting VEGF-A in myeloid cells enhances natural killer cell responses to chemotherapy and ameliorates cachexia
Source: Nat Commun. 2016 Aug 19;7:12528. doi: 10.1038/ncomms12528 (PMC4992172; doi:10.1038/ncomms12528)
Supplement: Supplementary Information — Supplementary Figures 1-10 [file ncomms12528-s1.pdf]

SUPPLEMENTARY FIGURE 1

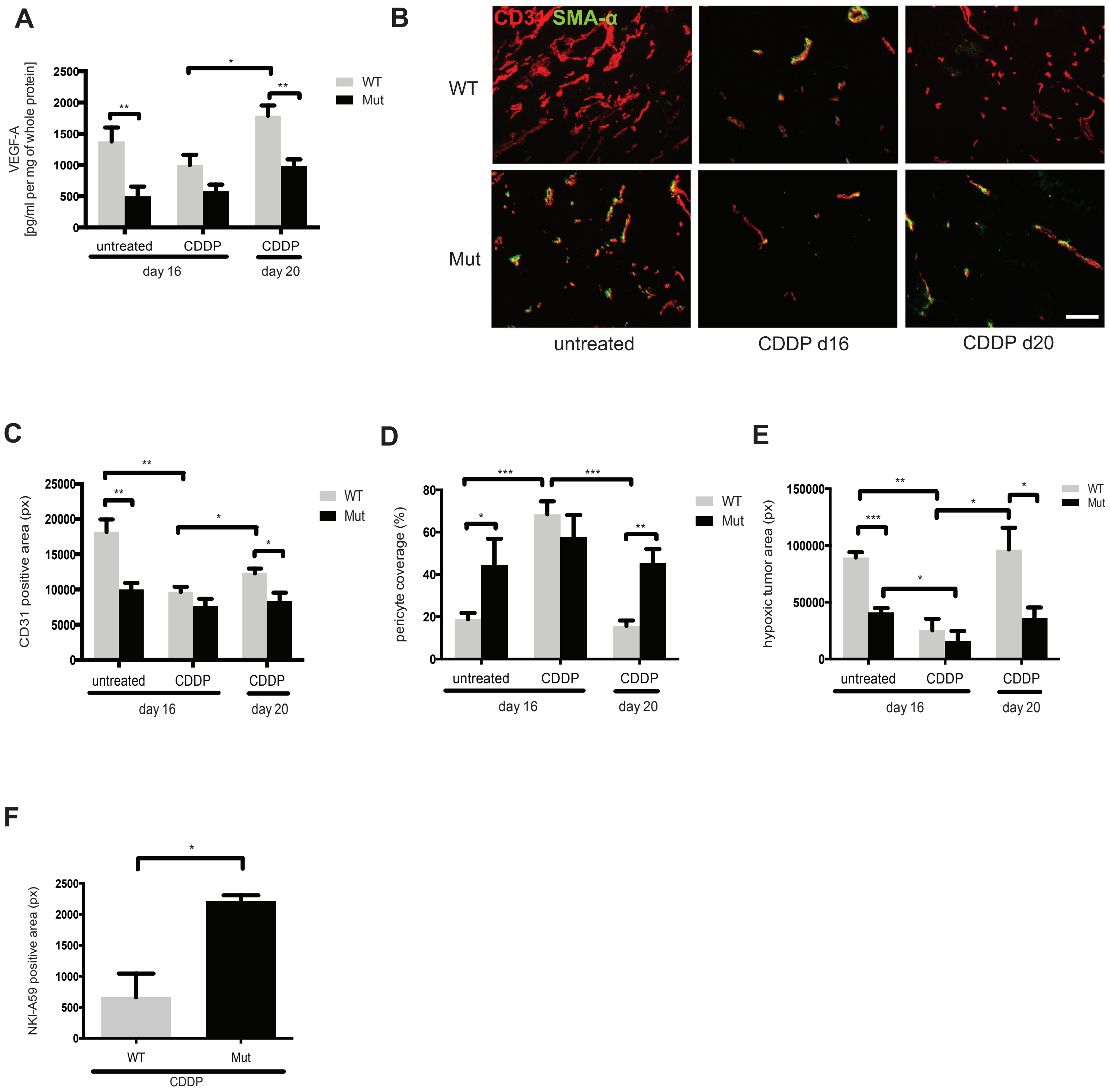

Supplementary Figure 1:

(A) Determination of levels of VEGF protein in B16F10 tumour lysates by ELISA (WT, n ≥ 4; Mut, n ≥ 4).

(B) Representative photomicrographs of co-immunolabeled CD31 and SMA-α B16F10 tumour sections from WT and mutant mice.

(C) Quantitative analysis of CD31 immunostaining shown in (B) (untreated, n ≥ 5; CDDP, n ≥ 6).

(D) Quantification of pericyte coverage as assessed by co-localization studies in (B). The fraction of pericyte coverage is given as the ratio of the number of SMA-α - to the number of CD31-positive cells (untreated, n = 4; CDDP, n ≥ 5).

(E) Quantification of pimodinazole-positive areas (untreated, n ≥ 5; CDDP, n ≥ 6).

(F) Quantification of cisplatin-DNA adducts on B16F10 tumour sections (day 16) (n=5).

SUPPLEMENTARY FIGURE 2

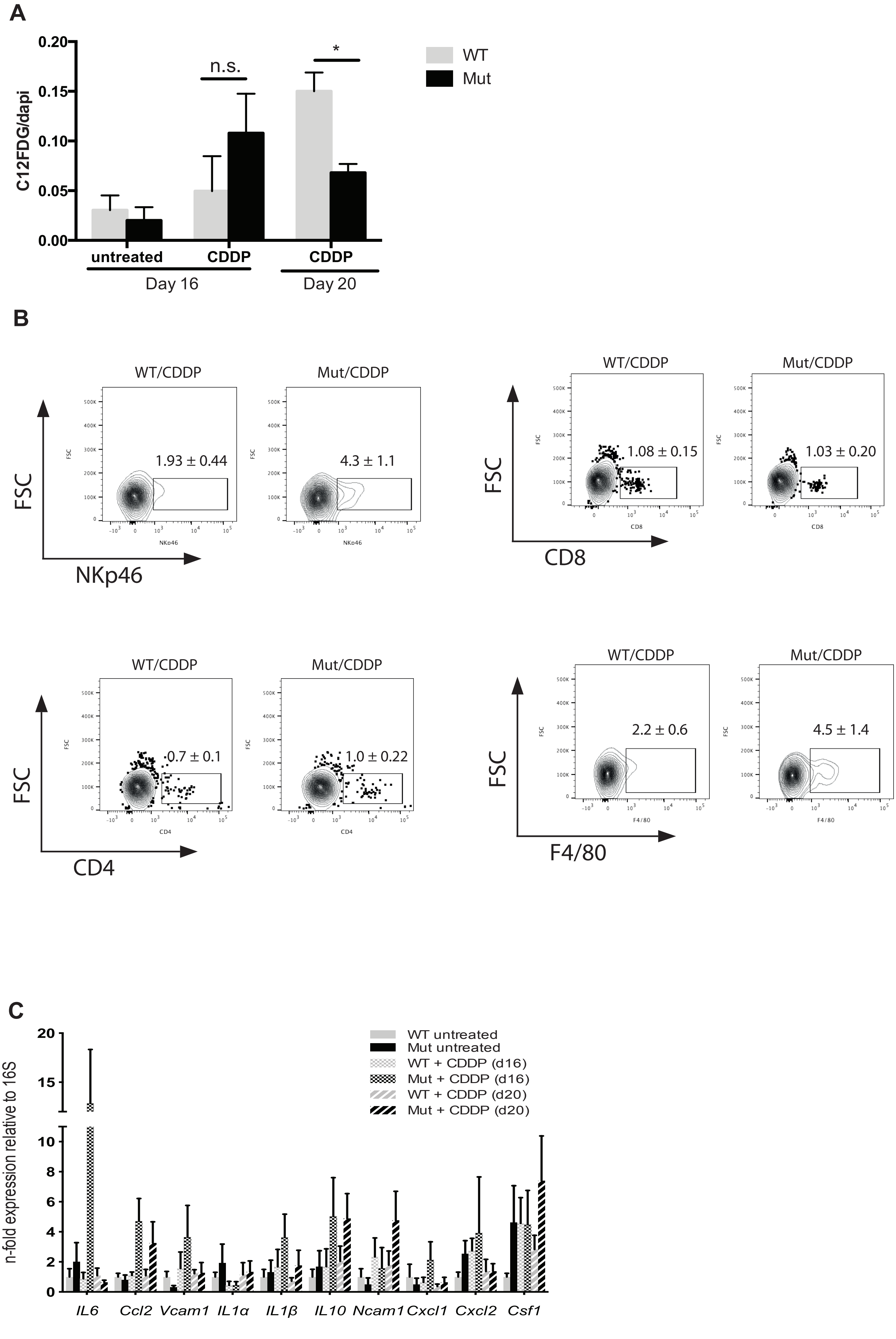

Supplementary Figure 2:

(A) Quantification of senescent C12FDG-positive cells on B16F10 tumour sections at indicated timepoints (untreated, $n \geq 5$; CDDP, $n \geq 6$).

(B) Flow cytometric analysis of cisplatin-treated B16F10 tumours showing percentages of tumour-infiltrating NKp46-, CD4-, CD8- and F4/80-positive cells among CD45 cells at indicated timepoints (representative of 3 independent experiments).

(C) N-fold change in gene expression of SASP factors in B16F10 tumours (untreated, $n \geq 5$; CDDP, $n \geq 6$).

SUPPLEMENTARY FIGURE 3

A

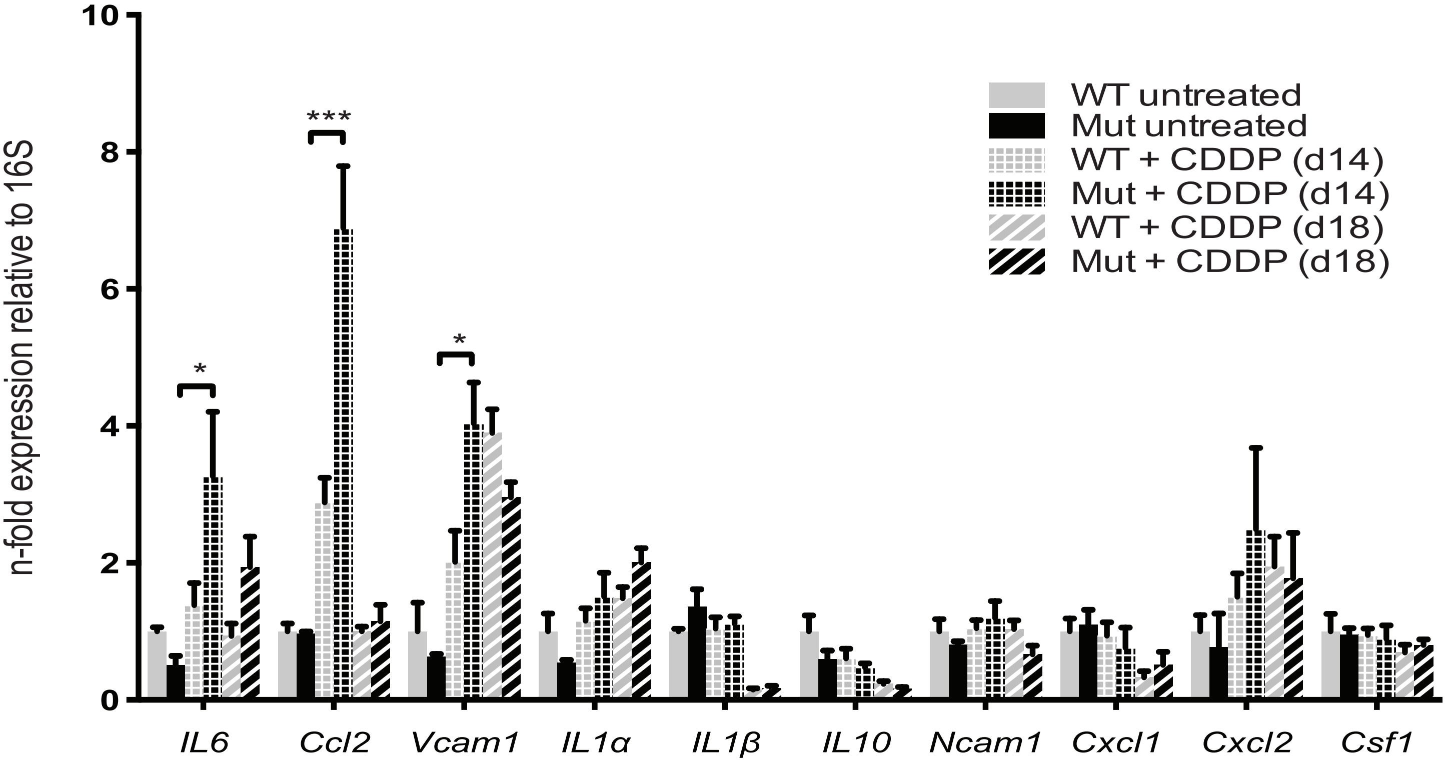

Supplementary Figure 3:

(A) N-fold change in gene expression of SASP factors in LLC tumours (untreated: n ≥ 4; CDDP: n ≥ 11).

SUPPLEMENTARY FIGURE 4

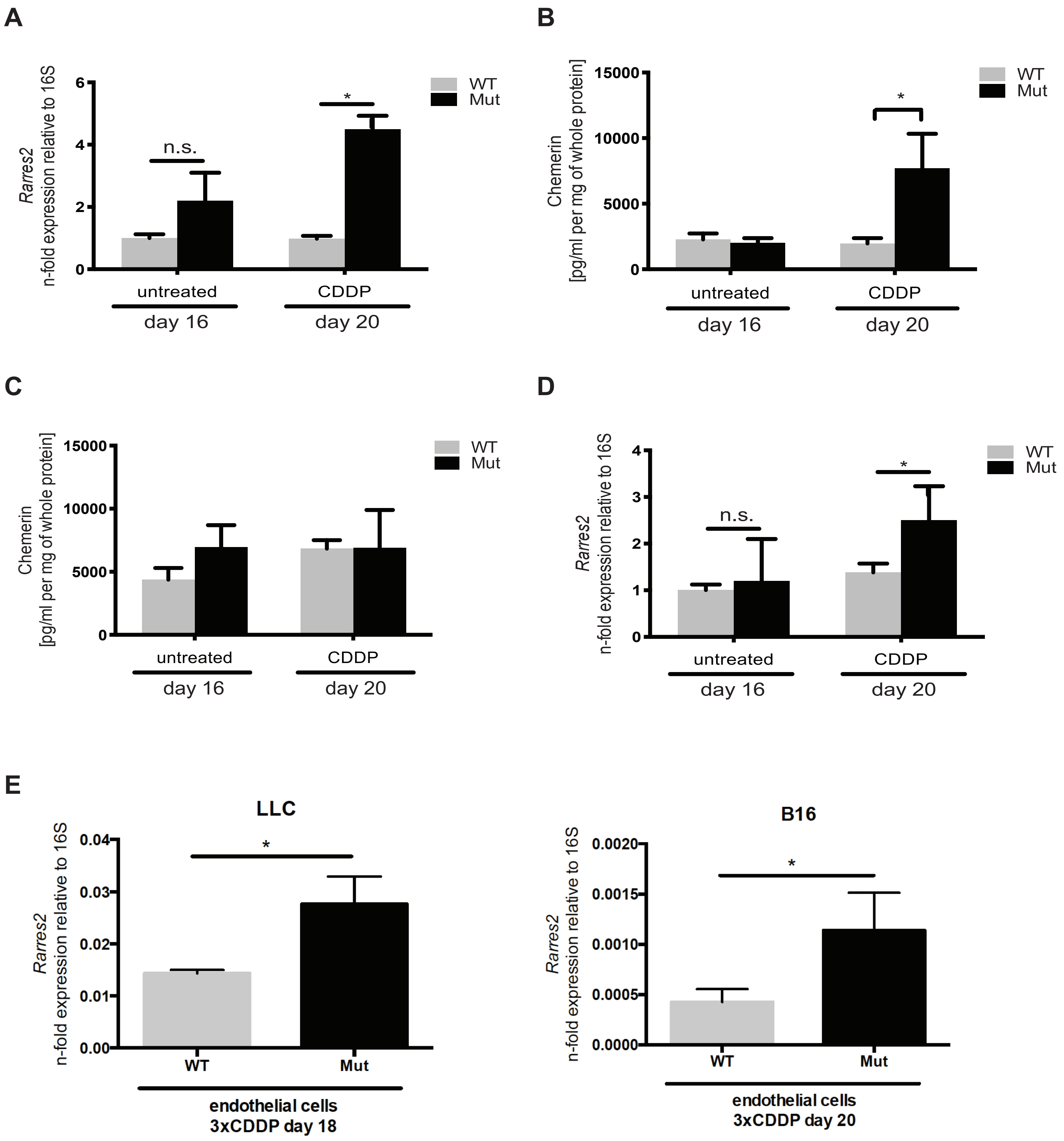

Supplementary Figure 4:

(A) Quantification of *RARRES2* gene expression by quantitative real-time analysis in B16F10 tumours at indicated timepoints (untreated, $n \geq 5$; CDDP, $n \geq 6$).

(B) Determination of levels of chemerin protein in B16F10 tumours at indicated timepoints (untreated, $n \geq 5$; CDDP, $n \geq 6$).

(C) Serum levels of chemerin in B16F10 tumours at indicated time points (untreated, $n \geq 5$; CDDP, $n \geq 6$).

(D) N-fold change in chemerin-expression of endothelial cells isolated from B16F10 tumours at indicated timepoints (untreated, $n \geq 5$; CDDP, $n \geq 6$).

(E) N-fold change in chemerin-expression of endothelial cells isolated from LLC (left) and B16F10 (right) tumours at indicated timepoints (CDDP, $n \geq 6$).

SUPPLEMENTARY FIGURE 5

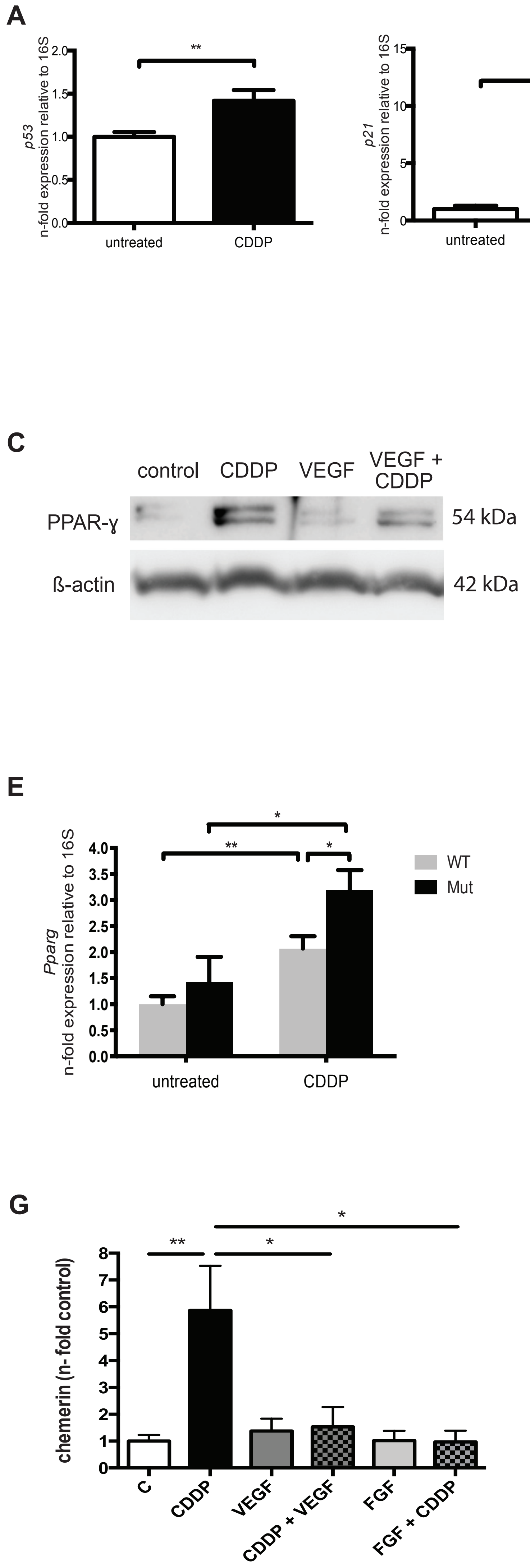

Supplementary Figure 5:

(A) N-fold change of *p53* and *p21* expression in *in vitro* cultured LLC tumour cells after treatment with CDDP (3 μg/ml) for 24 hours. Untreated cells served as control (n = 3).

(B) Quantification of chemerin protein levels in *in vitro* cultured LLC and B16F10 tumour cells treated with cisplatin (3 μg/ml) for 6 and 24 hours. Untreated cells served as control (n = 3).

(C) Representative Western blot of PPAR-γ expression in bEnd3 cells after cisplatin-treatment ± VEGF. ß-Actin served as loading control.

(D) Ratio of quantified PPAR-γ and ß-actin signals in (D) (n = 5).

(E) Quantitative real-time analysis of levels of *ppar-* γ transcripts in isolated tumour endothelial cells from LLC tumors at day 18 (untreated, n ≥ 4; CDDP, n = 7).

(F) Quantitative real-time analysis of levels of *ppar-* γ transcripts in isolated tumour endothelial cells from B16F10 tumours at indicated timepoints (untreated, n > 5; CDDP, n > 6).

(G) Quantification of chemerin protein levels of *in vitro*-cultured bEnd3-cells treated for 24 hours with cisplatin (3 μg/ml) or murine recombinant VEGF (25 ng/ml) or basic-FGF (10 ng/ml) or a combination of those. Untreated cells served as control (n = 3).

SUPPLEMENTARY FIGURE 6

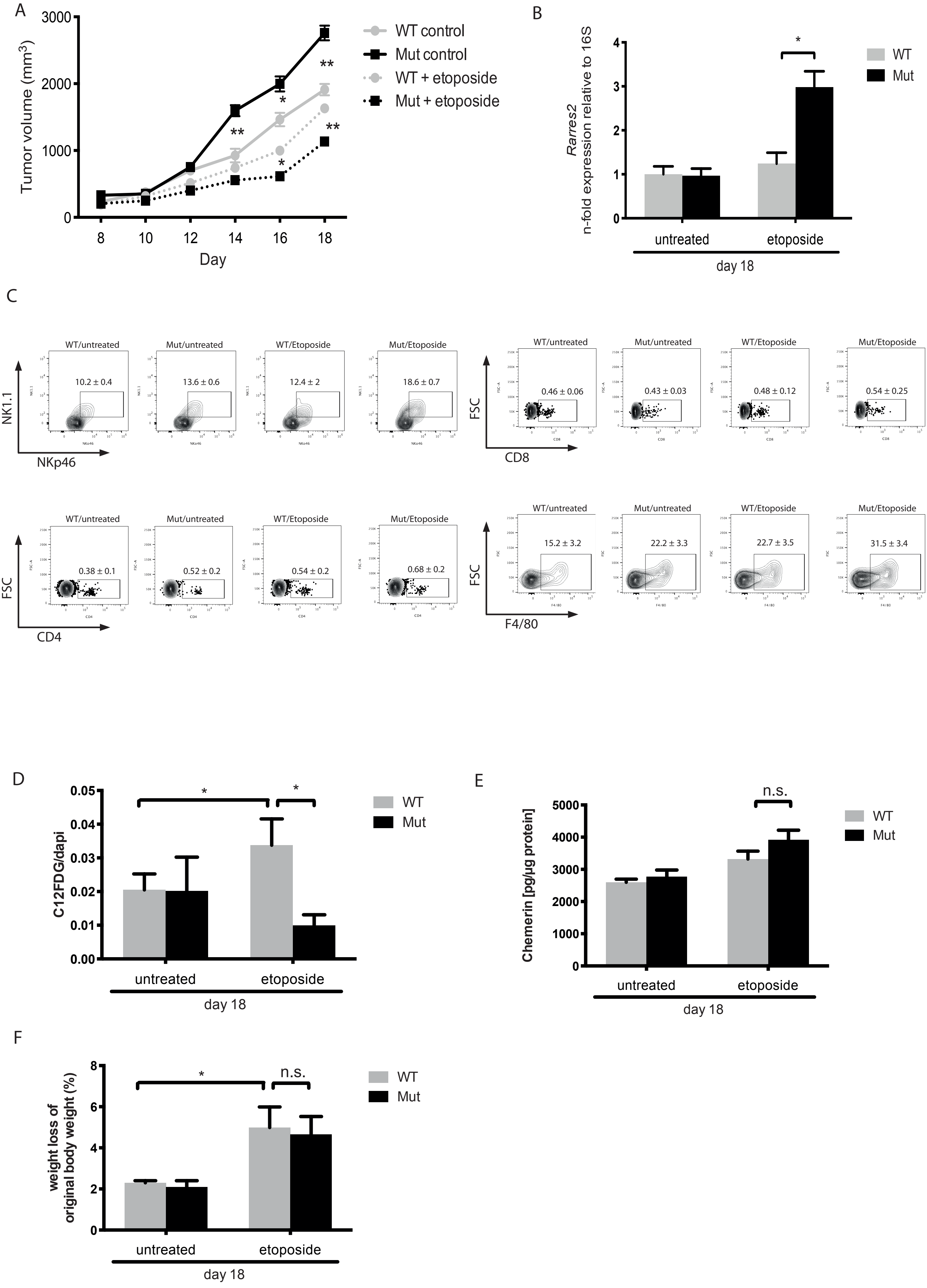

Supplementary Figure 6:

(A) Graphical representation of tumour growth kinetics of untreated and etoposide-treated (15mg/kg) LLC isografts after *s.c.* injection of tumour cells into WT or Mut mice (WT, n=8; Mut, n=6).

(B) Quantification of *RARRES2* gene expression by quantitative real-time PCR in endothelial cells (EC) isolated from etoposide-treated LLC tumours at endpoint day 18 (WT: n ≥ 5; Mut: n ≥ 5).

(C) Flow cytometric analysis of etoposide-treated LLC tumours showing percentages of tumour-infiltrating NKp46/NK1.1-, CD4-, CD8- and F4/80-positive cells among CD45 cells at endpoint day 18 (representative of 3 independent experiments).

(D) Quantification of C12FDG-positive cells on sections from etoposide-treated LLC tumours at day 18 (WT: n = 5; Mut: n = 5).

(E) Serum levels of chemerin of untreated and etoposide-treated LLC tumor-bearing mice at day 18 (WT: n = 5; Mut: n = 5).

(F) Body weight loss of untreated and etoposide-treated LLC-bearing mice at day 18. Weight loss is given as percentage of the original body weight (WT: n = 5; Mut: n = 5).

SUPPLEMENTARY FIGURE 7

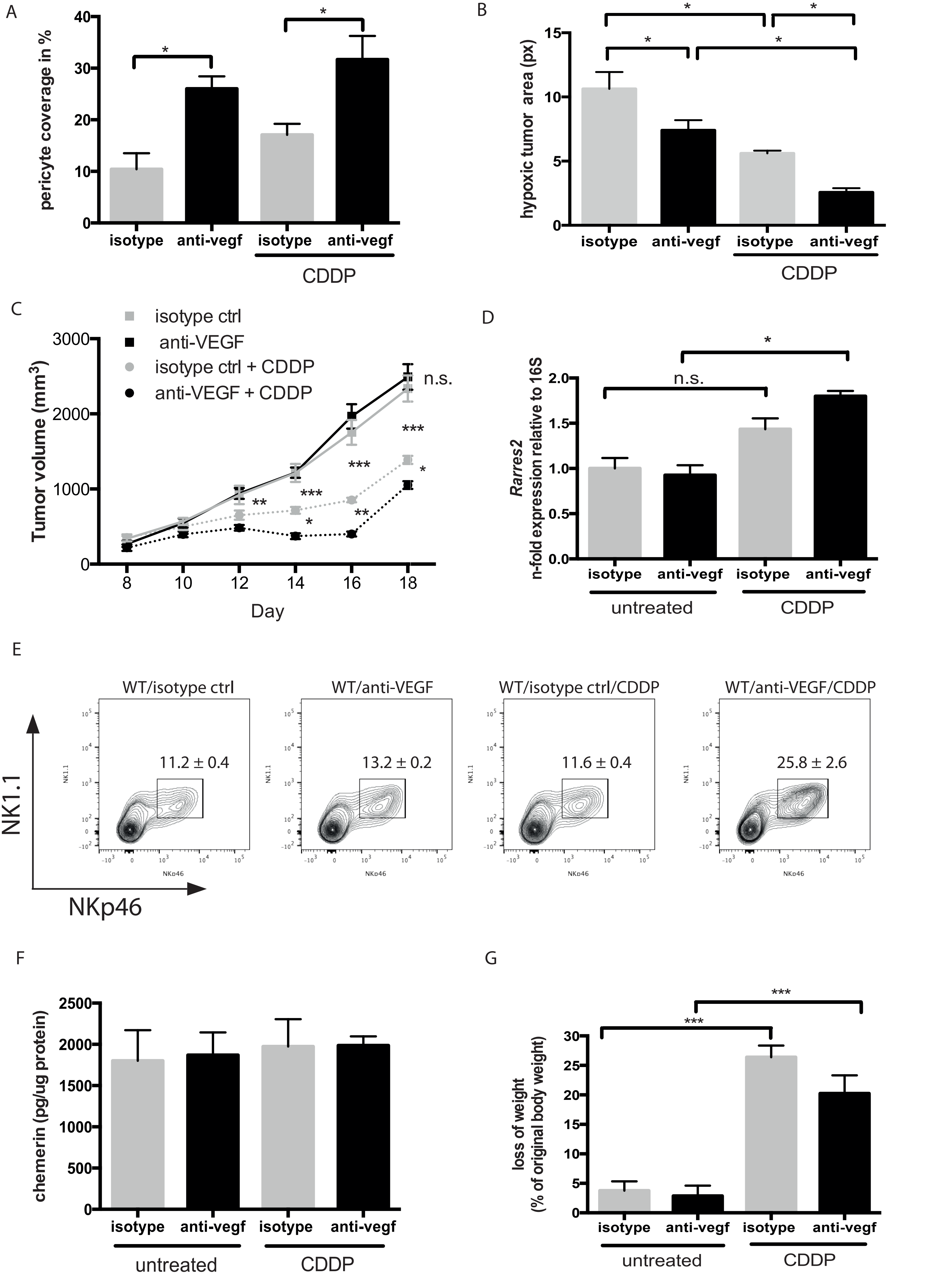

Supplementary Figure 7:

(A) Quantification of pericyte coverage in LLC tumours in C57Bl/6J mice treated with isotype antibody, anti-VEGF antibody and/or cisplatin at endpoint day 18 (isotype control, n = 10; anti-vegf, n = 11; isotype + CDDP, n = 6; anti-vegf +CDDP, n = 7). The fraction of pericyte coverage is given as the ratio of the number of SMA-α- to the number of CD31-positive cells.

(B) Quantification of hypoxic tumour areas in LLC tumours in C57Bl/6J mice treated with isotype antibody, anti-VEGF antibody and/or cisplatin at endpoint day 18 (isotype control, n = 10; anti-vegf, n = 11; isotype + CDDP, n = 6; anti-vegf +CDDP, n = 7).

(C) Growth kinetics of LLC tumours in C57Bl/6J mice treated with isotype antibody, anti-VEGF antibody and/or cisplatin (isotype control, n = 10; anti-vegf, n = 11; isotype + CDDP, n = 6; anti-vegf +CDDP, n = 7).

(D) N-fold change in chemerin-expression of endothelial cells isolated from LLC tumours in C57Bl/6J mice treated with isotype antibody, anti-VEGF antibody and/or cisplatin (isotype control, n = 10; anti-vegf, n = 11; isotype + CDDP, n = 6; anti-vegf +CDDP, n = 7) at endpoint day 18.

(E) Flow cytometric analysis of LLC tumours in C57Bl/6J mice at endpoint (day 18) treated with isotype antibody, anti-VEGF antibody and/or cisplatin, showing percentages of tumour-infiltrating NKp46/NK1.1-positive cells among CD45 cells at indicated timepoints (representative of 3 independent experiments).

SUPPLEMENTARY FIGURE 8

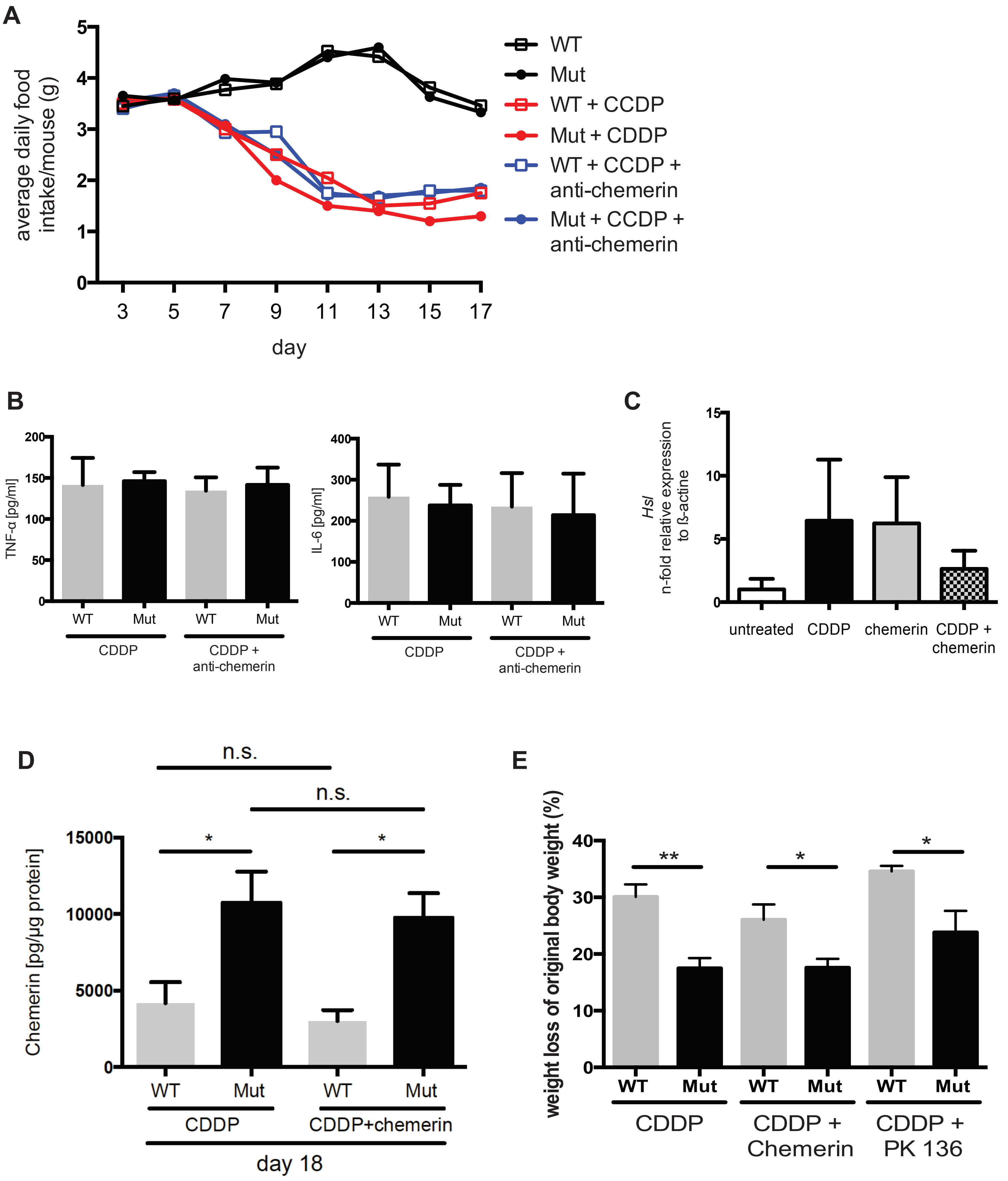

Supplementary Figure 8:

(A) Determination of average daily food intake of WT and Mut mice that received cisplatin-treatment with or without chemerin-neutralizing antibody. Black lines show intake of untreated LLC tumour-bearing mice (WT: $n \geq 4$; Mut: $n \geq 5$).

(B) Serum levels of TNF-α and IL-6 in WT and Mut mice after CDDP-treatment ± chemerin-neutralizing antibody (WT: $n \geq 5$, Mut: $n \geq 5$).

(C) Quantification of levels of *Hsl* transcripts in explant cultures of white adipose tissue from C57/Bl6J-WT mice. WAT explants were treated for 24 hours as indicated ($n \geq 4$).

(D) Serum levels of chemerin in WT and Mut mice after CDDP-treatment ± chemerin-neutralizing antibody at day 18 (WT: $n \geq 5$, Mut: $n \geq 5$).

(E) Body weight loss of LLC-bearing mice WT and Mut mice at day 18 after treatment with CDDP alone, CDDP and mAB PK136 or CDDP and intratumoral injections of recombinant chemerin ($n \geq 5$). Weight loss is given as percentage of the original body weight.

SUPPLEMENTARY FIGURE 9

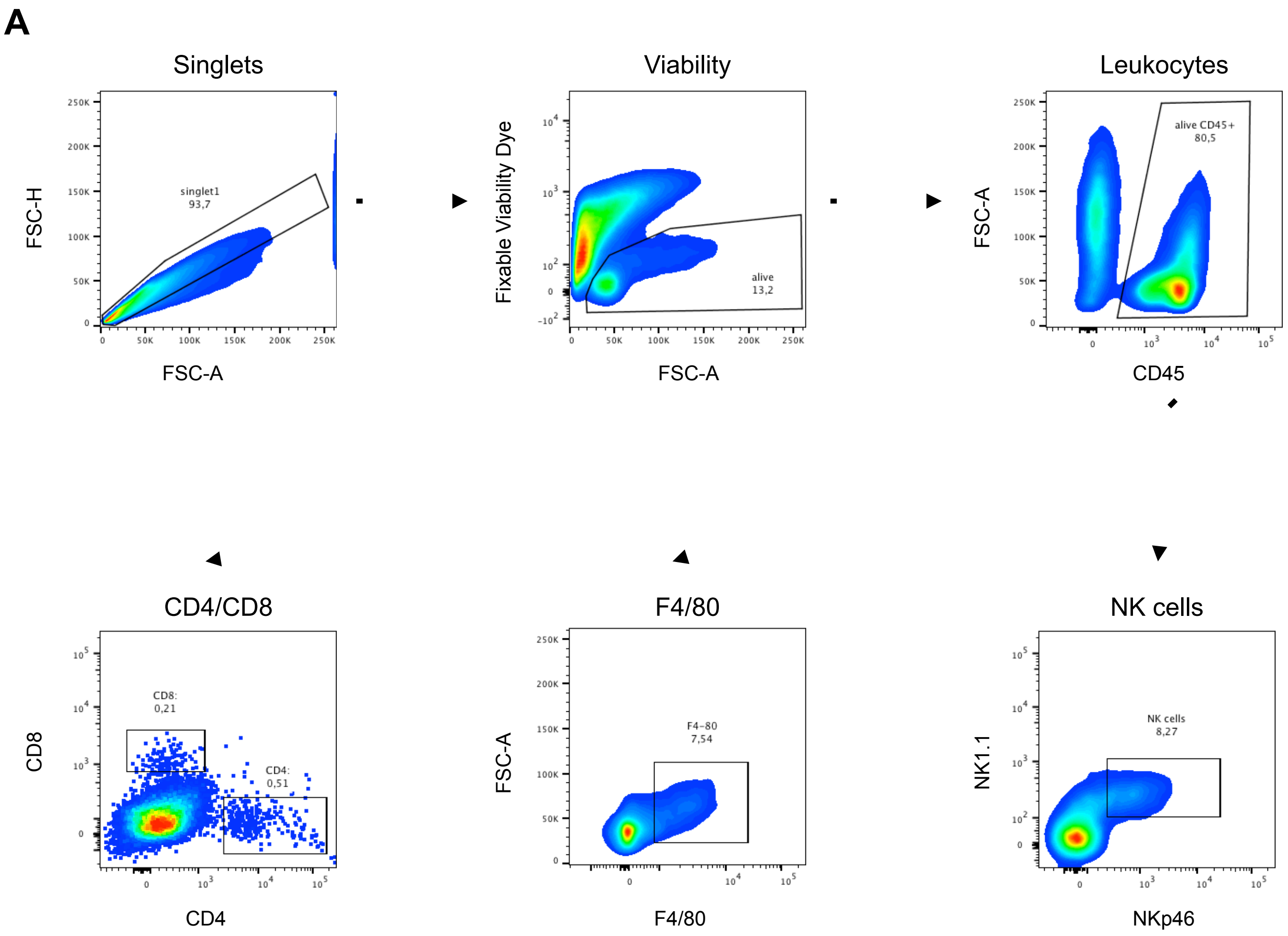

Abbreviations used:

Singlets (single events); NK cells (Natural Killer cells); F4/80 (Macrophages), CD4 (T helper cells); CD8 (T cytotoxic cells).

Supplementary Figure 9:

(A) Gating strategy: The single cell leukocyte population was selected by FSC-H versus FSC-A. The leukocyte population was further analysed for their uptake of the Live/Dead Aqua stain to determine live versus dead cells and for the expression of CD45. Then CD45+ cells were classified as NK cells by co-expression of NKp46 and NK1.1, macrophage population by F4/80 expression, T helper cells by CD4 expression and T cytotoxic cells by CD8 expression.

Supplementary Figure S5

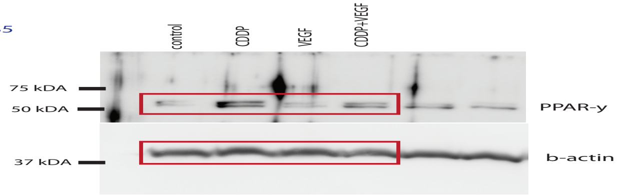

Supplementary Figure 10:

Scans of Western blots shown in Supplemental Figure 5C. Molecular weight markers are indicated. Red boxes highlight the lanes that are displayed in the corresponding Figures.

Bars represent mean values; error bars indicate the Standard Error of the Mean (SEM); * $p<0.05$, ** $p<0.01$, *** $p<0.001$, **** $p<0.0001$. Scale bar equals 100 μm.
